# Adaptive evolution of Pseudomonas aeruginosa ST299 population colonizing a hospital copper water network over a 2.5-year period

**DOI:** 10.1099/mgen.0.001585

**Published:** 2026-01-27

**Authors:** Maxine Virieux-Petit, Fabien Aujoulat, Chloé Dupont, Hélène Marchandin, Estelle Jumas-Bilak, Sara Romano-Bertrand

**Affiliations:** 1 HydroSciences Montpellier, Univ. Montpellier, CNRS, IRD, Service de Prévention des Infections et de la Résistance, CHU Montpellier, Montpellier, France; 2 HydroSciences Montpellier, Univ. Montpellier, CNRS, IRD, Montpellier, France; 3 MiVEGEC, Univ. Montpellier, CNRS, IRD, Laboratoire de Bactériologie, CHU de Montpellier, Montpellier, France; 4 HydroSciences Montpellier, Univ. Montpellier, CNRS, IRD, Service de Microbiologie et Hygiène hospitalière, CHU de Nîmes, Montpellier, France

**Keywords:** conserved genomes, copper resistance, patho-adaptation, *Pseudomonas aeruginosa*, successful colonization, whole-genome sequencing

## Abstract

**Background.** Descriptions of genomic characters and dynamics related to *Pseudomonas aeruginosa* (PA) adaptation and survival in hospital water networks remain scarce but necessary for sustainable water management in hospitals.

**Methods.** A new copper water network in an intensive care unit (ICU) was chronically colonized by a genotype sequence type (ST) 299 of PA and sporadically by a genotype ST2685. Sixty-eight ST299-PA and four ST2685-PA strains from ICU-water samples collected over 29 months and 4 months, respectively, were studied for genomic adaptation to copper water network. PFGE and whole-genome sequencing allowing SNP, comparative genomics, resistome, virulome and pangenome analyses were performed. In order to understand the adaptive phenomena linked to the colonization niche, 16 isolates of ST299-PA colonizing a cystic fibrosis (CF) patient during 16 months were included.

**Results and discussion.** The 68 ST299-PA ICU-water differed by <0.15 SNPs on average. No recombination regions nor patho-adaptive mutations were identified. Resistome, virulome and pangenome were stable over the time. The genomic content included a copper resistance operon, mainly metal resistance genes, a Tn*4661*-like transposon, the class 1 integron and broad-spectrum efflux pumps. These elements, absent in ST2685-PA ICU-water, support the survival of ST299-PA in the copper water network. The evolutionary speed of ST299-PA CF was faster with 12.9 SNPs among strains mostly affecting genes of patho-adaptation, arguing that the primo-colonization strain was probably not adapted to the niche, in contrast to the high genomic stability observed for the ST299-PA ICU-water population signifying the primary adaptation to the water network.

**Impact Statement**

The infectious success of *Pseudomonas aeruginosa* (PA) is well established, and PA can be described as a professional opportunistic pathogen. Identifying the genetic factors that contribute to PA adaptation in the hospital water network is essential for understanding its epidemiological success in healthcare-associated infections. Based on the local context of perennial colonization by PA, genotype ST299, for more than 2.5 years in the new copper water network in the medical intensive care unit, we found evidence of pre-adaptation of ST299 isolates and the presence of a metal resistance gene (MRG), GI-HMR, containing six genes involved in copper resistance. Metal resistance, and copper in particular, appears to be the cornerstone of this ecological success. The constraints of the water networks, and in particular the antimicrobial pressure exerted by copper, favour the adaptive evolution of PA, with the acquisition and development of skills (adaptive traits) ensuring its proliferation and long-term colonization of the water network. The presence of high copper levels favours the selection of copper-resistant bacteria and increases the permissiveness of plasmids carrying antimicrobial resistance gene and/or MRG. Copper resistance can be used by exaptation in other niches such as humans: this is the phenomenon of patho-adaptation, i.e. the use of a niche-specific adaptive trait to establish infection. The link between resistance to copper and antibiotics, on the one hand, and resistance to phagocytosis by macrophages or amoebae, on the other, leads us to reconsider the use of copper as an antimicrobial agent in the hospital environment.

## Data Summary

All genomic data were deposited in the NCBI under the project accession number PRJNA1064007. All supporting data are provided in supplementary data files. All accession numbers are given in Tables S1 and S3.

## Introduction

*Pseudomonas aeruginosa* (PA) is an ubiquitous saprophytic bacterium, capable to colonize a wide range of terrestrial, aquatic, anthropogenic and non-anthropogenic biotopes [1]. PA is also an opportunistic pathogen and a major causative agent of healthcare-associated infection (HCAI) [2,3], particularly in intensive care unit (ICU) or in immunocompromised patients [4]. According to the latest report from the European Centre for Disease Prevention and Control (ECDC) in 2019, 7.4% of patients who stayed in ICU for more than 2 days had at least one HCAI, with a majority of ventilator-associated pneumonia being caused by PA in 16.1% of cases [5].

PA can persist in water and plumbing systems as well as on wet surfaces and medical devices, making it an opportunistic premise plumbing pathogen, and has been described as highly adapted to the hospital environment [6]. The survival of PA within hospital niches is supported by its large core genome, which is particularly rich in adaptive genes, and its ability to form biofilm and resist major antibiotics and biocides [7,8]. Hospital water networks can therefore act as PA reservoirs where PA can not only persist but also multiply and thus represent a major source of PA transmission to patients [4,9,11]. Identifying the genetic factors that contribute to PA adaptation in the hospital water network is essential for understanding its epidemiological success in HCAI.

In hospital water networks, sink traps and U-bends are the preferred niches for the development of PA [12,14]. In this context, copper is used for water systems and as a coating agent on healthcare surfaces and medical devices due to its antimicrobial properties to limit bacterial colonization. Regarding PA, the survival of some genotypes, like sequence type (ST) 308 and ST395, within this environment has been incriminated in hospital outbreaks and shown to be facilitated by the acquisition of the GI-7 islet, which confers resistance to copper [13,15]. It has also been hypothesized that copper/antimicrobial co-selection promotes patho-adaptation and the emergence of hospital-successful PA genotypes [16].

The adaptive dynamics of PA has been described during persistence in the lungs of cystic fibrosis (CF) patients, the main study model to date. In CF respiratory tract, PA populations undergo genetic diversification over time driven by local selective pressures in the lung microenvironments and resulting in a heterogeneous population composed of clonally related variants favouring persistence [17]. Selective forces imposed by the host environment resulted in SNPs in regulatory/patho-adaptation genes responsible for convergent PA evolution [17]. The evolution and adaptation of PA have also been described during long-term colonization of the urinary tract [18]. In this context, SNP-mediated evolution occurred at a higher rate than that described during persistence in the CF airways. The SNPs impacted genes encoding transcriptional regulators and two-component systems, suggesting the key role of remodelling regulatory networks for PA adaptation to the urinary tract. Furthermore, bacterial persistence was associated with large deletion events resulting in convergent evolution of isolates in two patients. Thus, reduction of the catabolic gene repertoire, as previously described in CF, appears to be advantageous for PA adaptation to the human host [18].

To our knowledge, the adaptive evolution of PA in copper water networks has not been studied yet but could improve the understanding of the genomic traits that support PA survival and adaptation in such technological niches. The study was carried out in the context of the long-term colonization by PA (more than 2.5 years) of a newly built copper water network in a recent medical ICU [19]. This provides a privileged context to observe the adaptive phenomena occurring over time within the PA population in the water network. Therefore, the aim of this study was to determine whether the genomic traits and their evolution could explain the adaptation and persistence of a PA lineage of ST299 in the ICU-water network subjected to chronic stresses (exposure to copper and water chlorination) and episodic chemical shocks (decontamination cycles). We investigated the longitudinal dynamics of the virulome, resistome and pangenome of ST299-PA ICU-water strains and compared them with those of PA strains of other STs sporadically found in the ICU-water system. Moreover, the influence of the type of colonization niche was addressed by comparing the genomic evolution of ST299-PA strains from the water network and from the airways of CF patients.

## Methods

### Hospital setting and isolate collection

The medical ICU is organized in four distinct subunits according to the specificities of cares provided: the pink subunit (metabolic failures), the orange subunit (respiratory and infectious failures), the green subunit (haematological failures) and the blue subunit (burns patients). All subunits follow the same architectural scheme (Fig. S1, available in the online Supplementary Material) with a central space (CS) including a water point-of-use (WPOU) and five single rooms, each equipped with a WPOU. The water network supplying the medical ICU includes two independent loops: one loop supplying the pink and blue subunits and one loop supplying the green and orange subunits (Fig. S1). The strains collected from the water samples resulted from a second spray collected in a sodium thiosulphate-containing vial. The water sample was filtered through a 0.2 µm membrane immediately after collection. The membrane was then placed on Cetrimide agar and incubated at 30 °C for 48 h. If bacterial growth occurred, identification was carried out by MALDI-TOF. The PA communities were frozen in tryptone soy (TS) broth at −20 °C. The collection of strains collected from the water network was genotyped by multilocus sequence typing (MLST) according to Curran *et al*. [20]: 68 ICU-water strains belonged to the ST299, 4 strains belonged to ST2685 and 1 belonged to ST274 (Table 1).

**Table 1.** Antimicrobial resistance gene content determined by ResFinder and KmerResistance on a selection of 37 ST299-PA strains

Detected gene	Predicted phenotype	ST299-PA ICU (20 water strains and 10 clinical strains)	ST2685-PA ICU-water (*n*=4)	ST274-PA ICU-water (*n*=1)
*fos*A	Fosfomycin resistance	**+**	**+**	**+**
*cat*B7	Chloramphenicol resistance	**+**	**+**	**+**
*aph*(3′)-IIb	Aminoglycoside resistance	**+**	**+**	**+**
*crp*P	Ciprofloxacin resistance	**+**	**+**	**+**
*bla*_PAO_	*β*-Lactam resistance	**+**	**+**	**+**
*bla*_OXA-50_	*β*-Lactam resistance	**+**	**Ø**	**Ø**
*bla*_OXA-395_	*β*-Lactam resistance	**Ø**	**+**	**Ø**
*bla*_OXA-396_	*β*-Lactam resistance	**+**	**Ø**	**Ø**
*bla*_OXA-494_	*β*-Lactam resistance	**+**	**Ø**	**Ø**
*bla*_OXA-486_	*β*-Lactam resistance	**Ø**	**Ø**	**Ø**

+, Gene detected; Ø, gene not detected.

The 68 ST299-PA strains from ICU-water were collected within the 4 subunits over the period March 2015–July 2017, during 12 campaigns with 4 to 8 samples collected during each campaign (Table S1). Among the ST299-PA ICU-water isolates, 35 isolates were collected from WPOU within the blue and pink subunits (9 at the pink subunit CS and 8 in room 1; 5 at the blue CS and 12 in room 9) and 38 isolates from water points within the green and orange subunits (8 at the green subunit CS and 11 in room 13; 7 at the orange CS and 12 in room 17) (Table S2). The four ST2685-PAs were collected over the narrow May–September 2015 period, only in pink and orange subunits, and the ST274-PA strain was collected in July 2017 in the green subunit.

In collaboration with the medical bacteriology laboratory of the hospital, ten isolates of ST299-PA responsible for colonization or infection in six patients hospitalized in the medical ICU over the period of collection of environmental isolates, namely, ICU-clinical strain, were included in the working collection (Table S1). Sixteen ST299-PA strains collected from four sputum samples of a CF patient over the period May 2015 to November 2016 were also included in the study (Table S3).

### Antimicrobial susceptibility testing

The antibiotype (i.e. antibiotic resistance profile) of PA strains was assessed by the agar diffusion method, in accordance with the recommendations of the European Committee on Antimicrobial Susceptibility (EUCAST)[21]. The reference PA strain ATCC 27853 was taken as a control strain. The manipulations were carried out in a single replicate on all isolates.

### PA collection and genome sequencing

To sequence the PA isolates produced by storing the Cetrimide communities at −20 °C, an enrichment step was necessary: the PA communities were revived in TS broth at 37 °C for 24 h, and a culture was produced on TS agar. From this apparently homogeneous culture, which suggests the existence of a single morphotype, one colony per sample was sequenced. Consequently, diversity within samples was not studied during this work. Bacterial DNA from the 99 isolates was extracted from overnight cultures on trypticase soy agar using the MasterPure extraction kit (Epicentre). Within the ST299-PA population, the strains from ICU-water and ICU-clinical PA strains were first compared by multiplex rep-PCR according to Abdouchakour *et al*. [22] and PFGE with the use of *Spe*I or I-*Ceu*I enzyme (BioLabs New England) as previously described [23,24], while CF strains were typed by multiplex rep-PCR only. All isolates were sequenced by whole-genome sequencing (WGS) using Illumina NextSeq technology with an average coverage of 94.4% (ranging from 56 to 100%). The genome used is LTZ1. This low percentage of coverage is due to the alignment of PA isolates belonging to different STs (ST2685 and ST274) than the ST299 of the LTZ1 reference.

### Phylogenetic analysis: SNP calling

In order to obtain the dataset of core genome SNP (cgSNP), the ‘all-in-one’ variant calling tool Snippy (Galaxy Version 4.6.0+galaxy0) was used. The genome of the first isolated waterborne strain LTZ1 collected in March 2015 at the CS WPOU in the pink subunit was used as the reference genome. After ensuring the quality of the selected reference genome assembly (via alignment of LTZ1 reads against the LTZ1 assembly), it was annotated using PROKKA software (Galaxy Version 1.14.6+galaxy1). Genes and proteins impacted by the SNPs were identified. The occurrence of recombination events was assessed by Gubbins analysis (v3.2.1) [25]. Finally, a phylogenetic tree based on the cgSNP was generated using Seaview 5 software using the maximum-likelihood phylogeny method (PhyML v3.1) with the following parameters: general time reversible model and 100 bootstrap replicates. The remaining parameters were fixed by default.

### Resistome, virulome and pangenome

ResFinder v4.3.2 and KmerResistance 2.2 software were used to identify antimicrobial resistance genes (ARGs). ResFinder is based on the assembly of raw reads into contigs before comparing them to a reference database (detection via Basic Local Alignment Search Tool (blast) after assembly and then alignment) (Center for Genomic Epidemiology) [26]. The search of ARGs by KmerResistance is based on direct mapping of raw reads to reference sequences. ARG within an isolate can thus be detected without prior genome assembly by splitting the reads into k-mer and mapping them to reference databases (https://cge.food.dtu.dk/services/KmerResistance/) [27]. The identity threshold used by ResFinder to search for ARGs is 98%, which is considered optimal [28], whereas for KmerResistance, the search for ARG was performed using the default parameters.

In the absence of a database that would allow the determination of the PA mutational resistome, its evolutionary dynamics were assessed by searching for SNPs between pairs (including systematically the reference LTZ1 and reads of one strain of clade A). Secondly, the SNPs affecting genes considered as key in the modulation of the resistome were selected [29,30]. When an SNP affected a gene coding for a hypothetical protein according to the PROKKA annotation, a further blast search of the nucleotide sequences of the corresponding genes was conducted in the PseudoCAP database. This was done in order to produce a high-quality annotation of the PA genome [31] and to avoid any potential issues with missing key genes involved in the modulation of the resistome. Finally, synonymous SNPs were eliminated and non-synonymous SNPs selected.

The virulome was analysed using the virulence factor database (VFDB) [32] to extract the experimentally verified VF-encoding genes, i.e. VFDB dataset A corresponding to the virulome of PAO1 (NC_002516). The virulome of the isolates of the study was compared with those of three external strains of PA among the best characterized, namely, PA7 (NC_009656), UCBPP-PA14 (NC_008463) and PA-LESB58 (NC_011770). The blast Ring Image Generator (BRIG) tool was used to visualize the multiple genome comparison using the virulome of PAO1 as reference and where blast matches are coloured on a sliding scale indicating a defined percentage of identity of at least 80% (http://sourceforge. net/projects/brig/ ; V0.95-dev.0003) [33]. The presence/absence of virulence factors visible on the BRIG figure was verified via blast using the full PA virulence factor dataset available on the VFDB database, i.e. VFDB dataset B.

The search for mobile genetic elements (MGEs) such as integrons and transposons was carried out using BacAnt (https://github.com/xthua/bacant) [34].

To gain insight into the genome evolution in a PA population, a pangenome-wide study, more comprehensive than a study targeting virulome, resistome or SNPs, was essential. The principal benefit of a genome-wide analysis is that it obviates the necessity for the selection of a reference genome, thereby ensuring that the entire downloaded genomic content is integrated into the analysis rather than merely the regions identified in the reference.

The size of the pangenome collection and the core and accessory genes were determined using the pan genome pipeline Roary (v3.11.2) (http://sanger-pathogens.github.io/Roary) [35]. A pangenome analysis was conducted with the GView server (http://wishart.biology.ualberta.ca/cgview/) on a subset of ST299 ICU-water strains, with due consideration for their space-time distribution. Subsequently, the MAUVE alignment and visualization software (Mauve) was employed to ascertain the veracity of the genomic content differences, particularly to identify potential mis-assemblies in the contigs and to evaluate the presence or absence of genes split by the contigs [36].

## Results and discussion

### Pregenomic typing of the PA collection

The MLST typing of all PA strains collected in the ICU showed that the water network was persistently colonized by ST299-PA for more than 2.5 years: as 68 of the 73 ICU-water strains belonged to ST299. These strains were isolated from March 2015 to July 2017 and from the four subunits of the ward. Of the remaining five ICU-water strains, four belonged to ST2685, identified sporadically in the pink and orange subunits over a short period from May to September 2015, and one strain recovered in July 2017 was of ST274. All ST299-PA strains collected in the ICU, either from the water network or hospitalized patients (*n*=78), displayed an identical multiplex-rep PCR profile and the same profile A in PFGE (Table S1). Furthermore, examination of the *Spe*I and I-*Ceu*I pulsotypes indicates the absence of substantial genomic rearrangements and size variations. The rep-PCR and PFGE results suggested a genetic link between the 78 ICU isolates of ST299 [37]. Their genomes were sequenced to assess nucleotide polymorphism [38] and gene content.

Molecular typing by multiplex-rep PCR revealed that the 16 CF isolates exhibited diverse profiles, both between strains derived from disparate sputum samples and within strains derived from the same sample. This finding suggests the presence of significant genetic diversity among the isolates (Table S3). The whole-genome sequences of 16 CF isolates that displayed different rep-PCR fingerprints unrelated to those of the ICU strains were also determined in order to characterize the impact of the colonization niche. This was done by comparison of the genomic evolution of ST299-PA strains according to their origin.

### Phylogeny of ST299-PA population and origin of strains

**Fig. 1.**
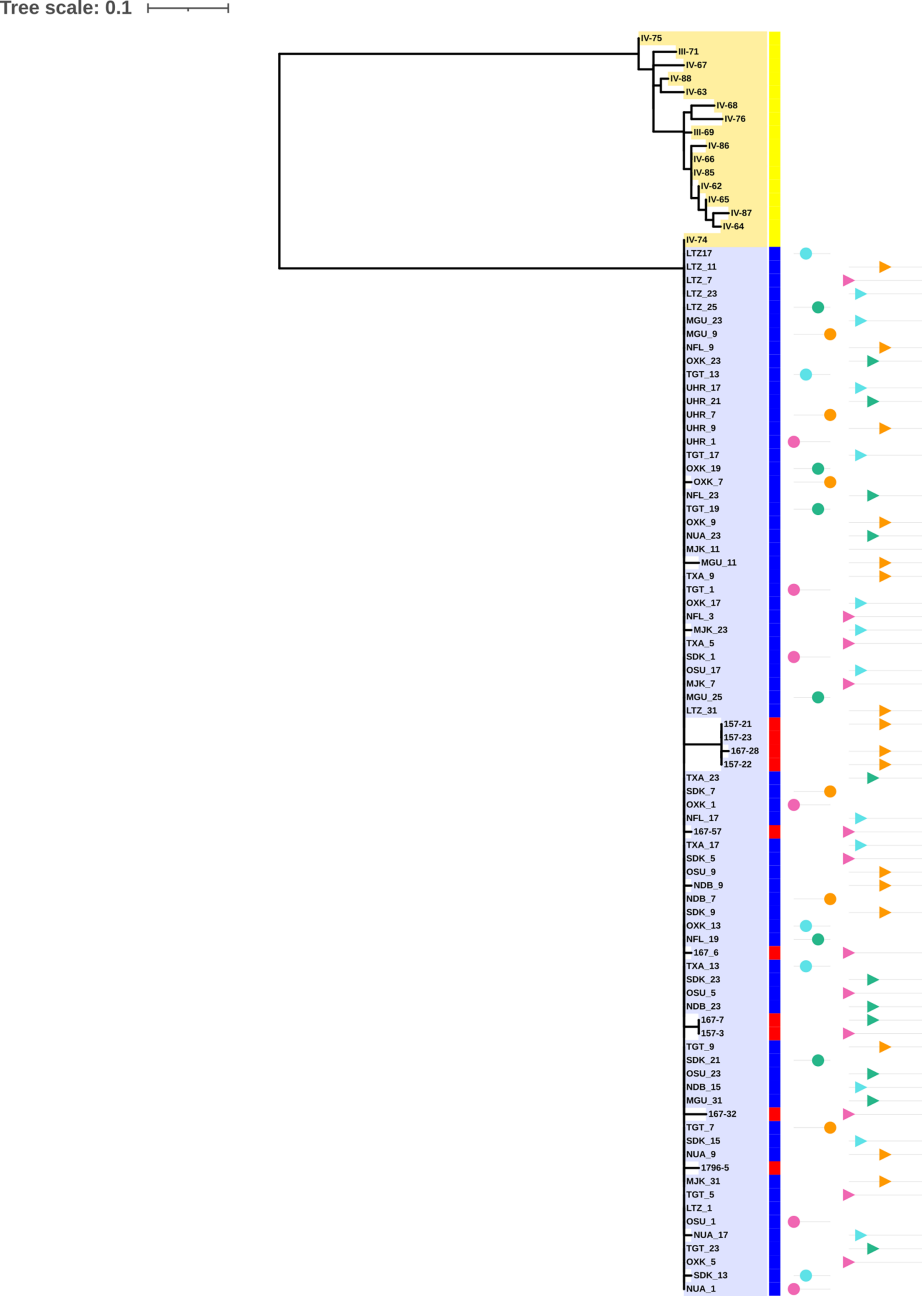
Maximum-likelihood phylogenetic tree based on the SNP of the core genomes of 94 ST299-PA isolates in the dataset (68 ICU-water isolates (blue), 8 ICU-clinical isolates (red) and 16 CF isolates (yellow). The cgSNP was obtained using the Snippy-core platform. Maximum-likelihood tree with 100 bootstrap replicates was reconstructed with SeaView 5. The online Interactive Tree Of Life (iTOL) tool was used to display and annotate the phylogenetic tree. The scale bar indicates the number of nucleotide substitutions within the cgSNP alignment. The phylogenetic tree revealed two clades designated clade A (highlighted in blue) and clade B (highlighted in yellow).

Phylogenetic analysis conducted on 94 ST299-PA strains (ICU and CF) was robust and showed two distinct clades named A and B (Fig. 1). The core-SNP alignment obtained using the Snippy core all-in-one variant calling tool was to 867 bp. After elimination of recombination regions via Gubbins, the core-SNP alignment (inter-clade SNP distance A-B) was of 111 cgSNPs. Two homologous recombination regions were identified between the ICU ST299-PA population and the CF ST299-PA population, both located on contig 1 of LTZ1 and measuring ~6,000 and 10,500 bp encoding *par*E1, *nik*R, *pil*T, *tcp*E, *sct*C and many hypothetical proteins.

Clade B gathered all the 16 CF strains collected from the same patient. Clade A clustered all ICU ST299-PA genomes (68 ICU-water strains and 10 clinical strains responsible for colonization or infection of patients hospitalized in the medical ICU) (Table S1). In clade A, the phylogenetic relationships between ICU-water isolates were independent of sampling date, subunit of care and type of WPOU sampled (CS or patient’s room).

The clustering of the ten ICU-clinical strains with all ST299 ICU-water strains within clade A, together with the detection of clade A-belonging strains in the water network before the first admission, suggests that ST299-PA infections occurring in ICU patients have a waterborne origin.

### High sequence conservation of ST299-PA ICU-water population

Core-SNP analysis restricted to clade A (water and ICU patient strains) highlighted the genetic conservation in clade A with an alignment of 25 cgSNPs, an average of 0.38 SNPs between genomes and no recombination regions. Among the 68 ICU-water-associated strains, most genomes (88.2%, *n*=60) displayed no cgSNP compared to the reference genome (profile 1), while the 8 remaining genomes each differed from the reference genome by a unique cgSNP profile (profile 2 to profile 9 with 1 or 2 cgSNP) (Fig. S2). Each cgSNP profiles 2 to 9 was found on single dates regularly distributed over the entire study period (8/12 sampling dates). Genomes of the ten ICU-clinical strains differed from the reference genome by one to six cgSNPs, underlining that most of the cgSNPs observed within clade A occurred in clinical strains (Fig. S2). In fact, core-SNP analysis restricted to ICU-water-associated strains revealed cgSNP alignment of 10 bp with an average of 0.15 cgSNP between genomes (Fig. S3).

Of the 25 cgSNPs identified in clade A, 23 occurred in 22 coding regions and included 6 synonymous mutations (low impact), 15 missense mutations (moderate impact but potential production of non-functional proteins) and 2 nonsense mutations in monocopy genes and two nonsense mutations (high impact), affecting a molybdenum transport protein and the transcriptional regulator *bkd*R of the Lrp family of proteins (Fig. S2).

The low level of SNPs within the genomes of clade A highlights the low genomic diversity and therefore the homogeneous population of PA strains that colonized the water network. To our knowledge, after ensuring the absence of methodological artefacts, this is the greatest genomic stability ever described for this species (supplementary data).

### Does the habitat of ST299-PA influence its genomic evolution?

To characterize the potential impact of the colonization niche on the evolutionary rate of ST299-PA, cgSNP analyses were performed independently on the ICU-water-associated strains (clade A), the ICU-clinical strains (clade A) and CF strains (clade B) (Fig. 1).

Due to the multiple selection pressures, especially antimicrobial but also inter-bacterial, applied to the lung tree of CF patients, a high rate of evolution of colonizing strains has been described. Pathoadaptive mutations are considered to be the main driver of evolution in the CF niche [39]. cgSNP analysis restricted to clade B, with the genome of strain III-69 corresponding to the first CF isolate collected in May 2015 as reference, reported no recombination events and showed a 46 bp alignment with an average of 12.9 cgSNPs/strain over the 18-month period of evolution in patient airways. Each isolate displayed a unique SNP profile: 16 different profiles were obtained for the 16 isolates (Fig. S4). The mean evolution speed of the PA clone is between 1.18 and 2.5 SNPs per year [40,42]. As expected, the rate of genomic evolution of the CF ST299 was high, giving rise to high genomic diversity among CF strains and favouring the emergence of subpopulations as described in CF [43].

Concerning the cgSNP analysis centred on the ICU-water-associated strains of clade A (LTZ1 as reference the genome and excluding human strains), we found a cgSNP alignment of 10 bp with an average of 0.15 cgSNP/isolate over the 27-month evolution and only 9 profiles among the 68 genomes with a majority profile identical to LTZ1 shared by 60 isolates (Fig. S4). We might also have expected a high rate of change in the ICU-water network submitted to various structural and chemical pressures, but this is not the case. However, when an ICU-water-associated strain moves on to humans, i.e. changes its niche, we see that it is able to adapt. In fact, the cgSNP analysis centred on the ICU-clinical strains of clade A (LTZ1 as reference the genome and excluding water strains) reported a cgSNP alignment of 22 bp with an average of five cgSNPs/strain (Fig. S5).

Concerning SNP selection, the ratio of non-synonymous substitution rate (dN) to synonymous substitutions rate (dS) is used to infer the type of selective force that shapes the population during adaptive evolution; a dN/dS ratio of greater than 1 suggests positive selection during evolution [44]. In this study, the ratio was 1 for ICU-water strains, 4.67 for ICU-clinical strains and 6.75 for CF strains.

So, for CF strains, almost all detected mutations are non-synonymous SNPs, and this provided the direct evidence that strong positive selective forces have dominated the PA genomes in the CF lungs. The SNPs affected genes in CF strains that were involved in patho-adaptation processes such as biofilm production (*alg*G), twitching motility (*pil*B, *pil*G and *pil*Z), regulation of metabolism (*nar*X), antimicrobial resistance (*mex*B and *amp*P) and acquisition of a mucoid phenotype (*muc*A).

Of the 25 cgSNPs identified in clade A, most among them were distributed in clinical strains with 15 cgSNPs of which 12 cgSNPs were missense mutations involved in patho-adaptation process such as regulation of metabolism (*atu*A), biofilm formation (*arn*B) and antimicrobial resistance (*arn*B, *mur*A, *mex*R and *amp*R) [45,47]. In contrast, among water strains, no SNPs were detected in any of the pathoadaptive genes described in PA including genes promoting adaptation to the specific niche represented by the water network (Fig. S4) [16]. Thus, SNPs among water strains did not reflect an adaptive response to known local constraints/pressures (copper, biocides, nutrient-poor environment, etc.) unlike the SNPs described in the genomes of PA strains isolated from ICU and CF patients. This result was previously reported in CF patient [48].

Finally, in ST299-PA, the evolutionary speed and positive selection were higher in CF and ICU patients than in water strains. This observation can be interpreted in terms of adaptation. It showed that the strain responsible for primo-colonization in CF and ICU patients was probably not adapted to the human host. For water strains, the value of the dN/dS ratio and no patho-adaptive mutations prove that SNPs were randomly distributed and exerted no discernible influence on the niche adaptation of ICU ST299-PA population. This result suggested a pre-adaptation of ST299 ICU population to the water network conditions.

### Small pangenome and large core genome in the ICU ST299-PA population

**Fig. 2.**
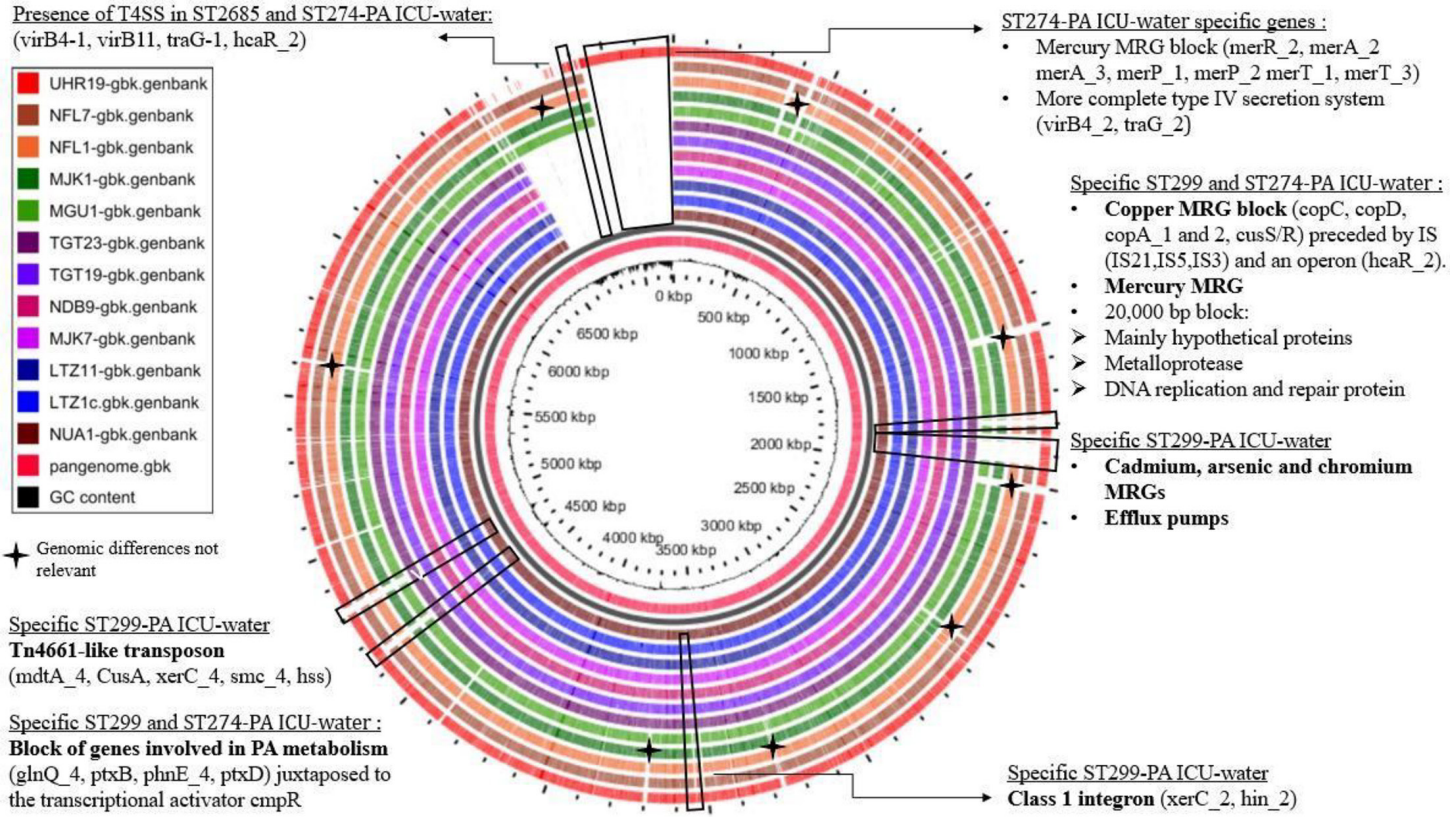
Analysis of the pangenome of 12 PA isolated from ICU-water samples over the study period (7 strains belonging to ST299, 4 strains to ST2685 and 1 to ST274). The figure was constructed by GView software. The innermost circle (pink) shows the pangenome constructed using all downloaded genomes. The following circles show the regions where there were blast matches between the constructed pangenome and the other downloaded genomes.

Pangenome analysis of the 78 ST299-PA ICU strains (water and human strains) revealed a total of 6,332 genes, of which 5,865 (almost 92.6 %) were common to ≥99% of strains (core genome). The core genome of PA was defined at 5,233 orthologues, which represented ~88% of the pangenome [49]. As the pangenome represents the cumulative genetic information in a set of genomes [50], the small size of the accessory genome attests to the strong relatedness in gene repertoire among strains and reflects the overall genomic conservation within ICU ST299-PA population. In order to assess the evolutionary dynamics of genome content and with regard to the genomic stability highlighted by the core-SNP, a pangenome analysis with GView was performed on a selection of 12 strains in the collection: 7 ST299-PA ICU-water strains covering the 2.5-year study period (Fig. 2) and all strains of ST2685 and ST274 (Fig. 2). The low number of SNPs identified, the absence of recombination events and the constancy of the gene repertory (Fig. 2) testify to the stability of the pangenome and suggest the ICU ST299-PA adaptation to local conditions/constraints.

Moreover, the stability of the pangenome underlies a low occurrence of horizontal gene transfer (HGT) events. This could be due to the low diversity of the associated microbial communities as observed by culture at the time of PA strain collection.

### Is the genomic content of ICU ST299-PA linked to copper water network adaptation?

#### Blocks of metal resistance genes, metabolic genes, integron and transposon

A block of metal resistance genes (MRGs) flanked by insertion sequences (ISs) (IS21, IS5 and IS3) was identified in WGS of all ST299-PA strains and ST274-PA (UHR19) but absent in all ST2685-PA strains (MGU1, MJK1, NFL1 and NFL7). This block carried six genes linked to copper resistance (*cop*C, *cop*D, *cop*A_1 and 2 and *cus*R and *cus*S genes of the CusRS two-component system) and two genes involved in mercury resistance (*mer*A and *mer*T). This heavy metal resistance unit is probably an operon governed by the transcriptional activator *hca*R located downstream of the first IS (IS3) and upstream of the MRGs. Juxtaposed to this copper resistance unit, there was a second block of MRG involved in resistance to cadmium (*cad*l), arsenic (*ars*C_1, *ars*C_2 and *acr*), chromium (*chr*A) [51] and two genes (*bep*E and *bep*F) coding for large substrate efflux pumps present only in PA-ST299 ICU strains. For the sake of clarity, we have decided to name the entire genomic block of MRGs, found only in PA-ST299 ICU strains, genomic island heavy metal resistance (GI-HMR). GI-HMR sized 99,152 bp with the inner block of the six genes involved in copper resistance sizing 10,145 bp.

In PA, a genomic island (GI) named GI-7 was described in the DH01 strain belonging to the successful ST395-PA genotype previously described as an epidemic high-risk clone [13]. The GI-7 island is a major determinant of copper resistance and plays an important role in the adaptation of bacteria to copper water systems. In hostile copper-rich environments, it allows the survival of PA by providing the bacterium the ability to adapt and colonize copper water systems [13,15]. The GI-7 island was absent in the ICU ST299-PA genomes. The alignment and comparison of the respective sequences of GI-7 island and GI-HMR showed alignments of 200 bp maximum. In order to assess the distribution of GI-HMR identified in the genomes of ICU ST299-PA strains, we searched the NCBI database, using the blast function (https://blast.ncbi.nlm.nih.gov). The ten genomes with the most significant alignments corresponded to seven PA strains, one *Bordetella genomosp.* strain, one *Xanthomonas arboricola* strain and one *Achromobacter xylosoxidans* strain, and all these bacterial species are of waterborne origin. For the seven PA strains, there were an average of 90,597 aligned bases for an average fragment size of 90,707 bp with more than 99% homology with GI-HMR sequence. The seven PA matching sequences belonged to diverse PA genotypes: ST1248 (*n*=1), ST998 (*n*=1), ST260 (*n*=3) and ST299 (*n*=2). The PA strains were derived from clinical samples (6/7), of which half (3/7) were collected from CF patients, and the origin of one of them was unknown. This suggests that GI-HMR was frequently present in the PA species and especially in strains of ST299 and may be essential for the adaptation of ST299-PA to the copper water network. Compared to both ST2685-PA and ST274-PA ICU-water, ST299-PA ICU-water strains displayed other specific MGEs: a Tn*4661*-like transposon and a class 1 integron. The Tn*4661*-like transposon carried three genes that may be advantageous for the survival of PA within the water system, namely, the *mdt*A and *cus*A genes involved in antimicrobial and metal resistance, respectively, as well as the *smc* gene encoding a condensin involved in the control of multiple genetic programmes related to the epigenetic and virulent behaviour of PA [52]. On the other hand, integrons represent versatile gene acquisition systems and a source of genomic complexity that shape phenotypic diversity and adaptive responses. Thus, the presence of the In498 integron within the genomes of ST299-PA ICU-water strains can confer an adaptive advantage in the face of environmental constraints in the water system, in spite of an unclear gene content, consisting mainly of hypothetical proteins and the *xer*C and *hin* genes. The *xer*C gene is frequently found in close proximity to plasmid-transmitted resistance genes and is thought to facilitate recognition and integration into the host [53, 54]. The *hin* gene codes for an ADN invertase protein and is involved in the formation of the recombination complex [55]. A block of genes involved in PA metabolism (*gln*Q, *ptx*B, *phn*E and *ptx*D) juxtaposed to the transcriptional activator *cmp*R was also specifically observed in the WGS of both ST2685-PA and ST274-PA ICU-water strains.

Finally, it is likely that all these elements specifically present in ST299-PA ICU-water genomes favour the fitness of this population facing the environmental constraints in the ICU-water network. ICU-water PA strains of ST2685 and ST274 did not colonize perennially the water system (Table S1) and did not carry these genetic elements, suggesting that their transitory presence was linked to a less adaptive ability conferred by their genome content.

#### Stable and rich/dense virulome

**Fig. 3.**
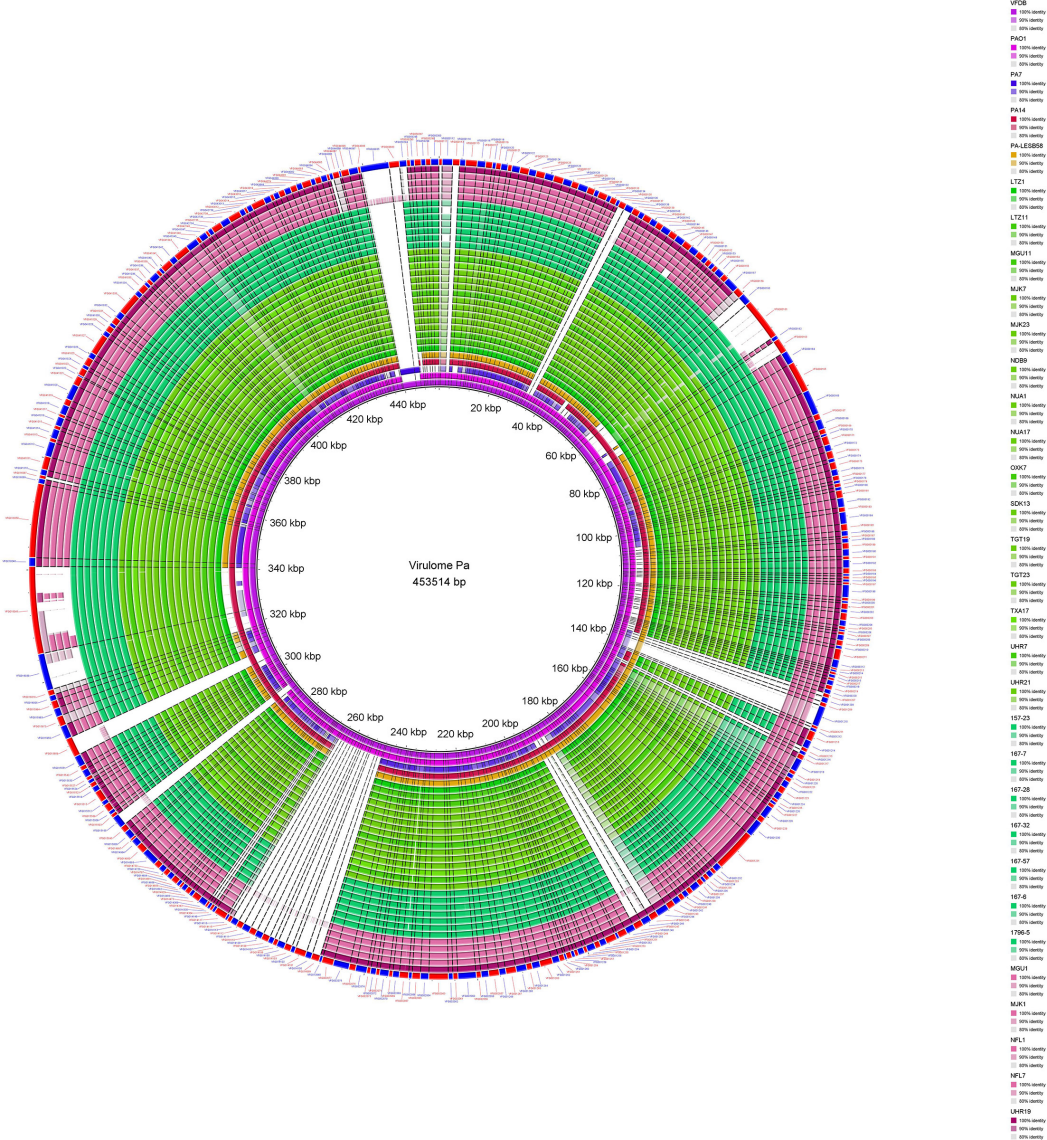
Virulome analysis for 15 ST299-PA ICU-water, 4 ST2685-PA ICU-water, 1 ST274-PA ICU-water and 7 ST299-PA ICU-clinical strains. Circular representation of a panel of genomes of 22 strains studied and 4 PA reference strains (PAO1, PA7, PA14 and PA-LESB58). The draft genomes and the 4 reference genomes were aligned with the theoretical PA virulence pangenome constructed from the VFDB extraction of the 324 virulence genes of the PA species and verified experimentally. Each reference genome is represented by a different coloured ring (pink for PAO1, blue for PA7, red for PA14 and orange for LESB58). The ST299-PA ICU is represented by a green ring, light if of water origin and dark if of clinical origin. Similarly, the set of genomes no ST299-PA ICU-water is represented by a pink ring, light for the four isolates ST2685 and dark for the ST274 isolate. The outermost circle, alternating red and blue, represents the virulence genes. The colour gradient indicates the percentage of identity of the virulence gene in the genomes studied in relation to the theoretical virulence pangenome of PA. The image was generated using BRIG.

The virolome and resistome were determined on a sub-collection of isolates that met the criteria for coverage of all cgSNP profiles and that were distributed uniformly in space and time. This ensured that the analysed sample was representative of the studied collection. The presence of a dataset of 324 virulence genes of PAO1, *exo*U gene of PA14 and *exl*A and *exl*B genes of PA7 was analysed in 22 ST299-PA genomes (including 15 genomes of ICU-water strains and 7 genomes of clinical ICU strains), all isolates ST2685-PA (*n*=4) and the strain ST274-PA (Fig. 3). These virulence genes are associated with lagella and pili biosynthesis/regulation, protease production, type II, IV and VI secretion systems, protease IV, enzyme, quorum sensing, alginate production/regulation, and toxins were curated from the VFDB.

Firstly, the virulome in ICU ST299-PA selected population was stable as no evolution was observed over the 2.5-year follow-up period. Secondly, ICU ST299-PA strains appeared to be rich in virulence genes as they possessed the majority of virulence genes described in PAO1 (Fig. 3).

*A priori*, a block of genes involved in type IV pili synthesis (*pil*A, *pil*V, *pil*W, *pil*X, *pil*Y1, *pil*Y2, *pil*E, *fim*U and *fim*T) and a block of genes involved in flagellum synthesis (*flg*K, *flg*L, *fli*D, *fle*I, *fli*S and *fli*T) appeared to be absent in ICU ST299-PA but in fact ST299-PA had different alleles of these genes than PAO1, explaining the bias produced by BRIG analysis [56]. The virulence gene content appears to be comparable between the successful ST299-PA population and the ST2685-PA population present sporadically in the water network, and the virulome therefore cannot explain the success of ST299-PA in this technologically driven ecological niche.

#### Stable resistome during persistence in the copper water network

The search for ARGs was conducted on 35 PA strains including 30 ST299-PA strains collected from the water network and patients in the medical ICU and selected to cover all SNP profiles and respect a uniform spatiotemporal distribution in order to be representative of the entire collection, the four ST2685-PA strains and the ST274-PA strain. The core resistome detected in all the 35 genomes consisted of the six following ARGs: *fos*A encoding fosfomycin resistance, *cat*B7 encoding resistance to the phenicol class, *aph*(3′)-IIb encoding resistance to aminoglycosides, *crp*P encoding resistance to ciprofloxacin, *bla*_PAO_ and an oxacillinase-encoding *bla*_OXA-50_-like gene (differing by two to ten nucleotides from the 789 bp reference *bla*_OXA-50_ sequence (accession number AY306130.1) likely involved in *β*-lactam resistance. Antimicrobial susceptibility testing was conducted on ST299-PA ICU-water strains and revealed a stable resistance profile over a period of almost 2.5 years and a resistance phenotype consistent with the genotype, indicating no enrichment of resistance genes by HGT over time (Table S4). This observation may seem surprising in the light of the known versatility of PA and hospital environmental pressures exerting in water plumbing systems favouring HGT phenomena [57,58]. Indeed, in addition to sub-inhibitory concentrations of antibiotics, non-antibiotic pharmaceuticals like ammonium compounds and trihalomethanes can also promote antibiotic resistance through HGT [58].

Regarding the mutational resistome of ST299-PA ICU-water, a missense mutation occurred in the *fol*D gene of two strains. Such a mutation in the *fol*D gene can result in an up-regulation of the expression of the MexXY-OprM RND efflux pump [59], which is widely implicated in aminoglycoside resistance. In the present case, no modification in the aminoglycoside resistance phenotype was observed in both strains, already displaying *aph*(3′)-Iib-encoded aminoglycoside resistance. However, due to the phenomena of cross-resistance [16], the RND efflux pumps like MexXY-OprM are involved in copper resistance [60], so the mutation *fol*D can be considered as a patho-adaptative mutation. Except for this mutation, no SNPs were identified among the 164 genes involved in modulating the resistome for other ST299-PA ICU-water strains. For ICU-clinical strains, further SNPs affecting genes relevant for the modulation of the resistome were identified (Table 2), in agreement with their resistance to piperacillin and ticarcillin and their respective association with tazobactam and clavulanic acid, found in clinical isolates only, whereas absent in ICU-water isolates.

**Table 2.** Mutational resistome within clade A strain genomes

	Mutated gene	Possible effect	Acquired resistance	Reference
ICU-water strains (*n*=68)	*fol*D	Up-regulation of expression of the RND pump	Aminoglycoside resistance	[59]
ICU-clinical strains (*n*=10)	*amp*R Transcriptional regulator	Hyperproduction of the cephalosporinase AmpC	Resistance to almost all *β*-lactams, except carbapenems	[30,71]
		Control of quorum sensing (QS)-dependent VF production, biofilm and alginate production: role in the transition from acute to chronic infection		[72,73]
	*opr*D	Inactivation of OprD porin	Carbapenem resistance	[74,75]
	*mex*R	Overproduction of the MexAB-OprM RND efflux pump	Resistance to almost all *β*-lactams (except imipenem) and resistance to fluoroquinolone	[30,76]
	*opr*M Outer membrane component of the MexAB-OprM efflux system	Possibly no effect: a mutation in conserved regions of the protein does not always result in loss of function of the RND pump		[77]

## Conclusive discussion

In the context of a persistent colonization of a newly built copper water network of an ICU by a single genotype of PA, ST299, over a period of 2.5 years, we showed that the ST299 lineage (clade A) is involved in the perennial colonization of the water network, while other PA strains did not succeed in long-term water network colonization. This suggests a pre-adaptation of ST299 clade A within this technologically driven niche rather than the genetic diversification of the non-fully adapted initial population as described in CF [43].

The genomic stability of ST299-PA during 2.5 years despite variations in the pressures exerted on its niche (repeated shocks of different kinds, changes in practices) [19] contrasts with the literature that describes the genome of PA as polymorph and flexible [61,62]. Such a surprising result required excluding methodological biases (supplementary data).

Phylogenomics, virulome and pangenome analyses of 78 ICU ST299-PA strains, either of water or clinical origin, showed a population very homogeneous and preserved. Regarding the resistome, it was stable during persistence in the copper water network, but when the ecological niche changes, with the transition to man, punctual mutations are observed involved in genes modulating the resistome and seem to be directly correlated with the evolution of antimicrobial resistance phenotypes revealed by antibiograms.

The large core genome, the small accessory genome, the low level of SNPs showing a slow rate of evolution, the ratio dN/dS showing no positive selection of SNPs and absence of patho-adaptative mutation during the entire period of colonization of the ICU-water network reflect the primary adaptation of the ST299-PA population to this technological niche and suggest that the copper water network of the medical ICU was surely contaminated by ST299-PA before its opening or even at the time of its construction as already reported by Petitjean *et al*. [63]. A Bayesian analysis would have permitted the estimation of the emergence of the PA lineage of ST299 at the hospital [63].

The accessory genomes are usually composed of horizontally transferable elements which include integrative and conjugative elements, GIs, prophages, transposons, ISs and integrons [64], and they often contribute to the unique physiology, pathogenesis and antibiotic resistances of the corresponding strains [65,66]. Analysis of the ST299-PA ICU-water population identified a GI, GI-HMR, never before described in the literature, present in several proteobacteria species but more frequently in PA and especially the genotype 299. This island contains six copper resistance genes, multiple ARGs and large substrate efflux pumps and, like the GI-7 island, could give ST299 a selective advantage for persistence in a copper water network and for patho-adaptation [13,16]. The six genes involved in copper resistance participate in the optimal management of intracellular copper concentrations in order to counteract the toxicity of this element and participate in tolerance of ST299-PA ICU-water strains to the copper pipe walls [16]. Due to the now clearly demonstrated copper/antimicrobial co-selection phenomenon, the presence of GI-HMR may be beneficial to resist antimicrobial pressure, through mixed efflux systems or co-regulation processes [16]. Moreover, copper and arsenic resistance in PA play a crucial role in bacterial resistance to amoeba predation [67,68], which is particularly prevalent in hospital water ecosystems [69,70]. Thus, the survival of copper- and arsenic-tolerant bacteria in water distribution systems may be enhanced by their increased resistance to amoebic predation, i.e*.* resistance to intra-amoebic digestion. The ICU-water ST2685-PA strains sporadically found in the water network seem to be less equipped than their ST299 counterparts to cope with environmental stressors common to hospital water networks such as copper, antimicrobials, biocides or amoeba predation.

The pre-adaptation discussed above and the GI-HMR certainly contributed to the strain’s survival in the copper water network. However, no genomic event seems to explain the sudden decline of ST299-PA after 2.5 years of water network colonization, demonstrating the complexity and limitations of using genomics alone and the need to combine it with broader studies including protein, metabolomics and phenotypic studies. Furthermore, the comparative study of ST299-PA from two different niches, the lungs of CF patients and the water network, showed that the rate of genome evolution and the resulting adaptive mechanisms are dictated by the specific ecological constraints. To our knowledge, we described herein one of the more stable populations of PA during a long-term follow-up despite selective pressure and biocide usage in hospital environment.

## Supplementary material

10.1099/mgen.0.001585 Uncited Fig. S1.

## Abbreviations

ARG: antimicrobial resistance gene
blast: Basic Local Alignment Search Tool
BRIG: blast Ring Image Generator
CF: cystic fibrosis
cgSNP: core genome SNP
CS: central space
GI: genomic island
GI-HMR: genomic island heavy metal resistance
HCAI: healthcare-associated infection
HGT: horizontal gene transfer
ICU: intensive care unit
MGE: mobile genetic element
MLST: multilocus sequence typing
MRG: metal resistance gene
PA: *Pseudomonas aeruginosa*
ST: sequence type
VFDB: virulence factor database
WGS: whole-genome sequencing
WPOU: water point-of-use

## References

[1] Silby MW, Winstanley C, Godfrey SAC, Levy SB, Jackson RW (2011). *Pseudomonas genomes*: diverse and adaptable. FEMS Microbiol Rev.

[2] Kucisec-Tepes N (2004). *Pseudomonas aeruginosa*--a significant hospital pathogen and resistance to carbapenem. Acta Med Croatica.

[3] Lambert M-L, Suetens C, Savey A, Palomar M, Hiesmayr M (2011). Clinical outcomes of health-care-associated infections and antimicrobial resistance in patients admitted to European intensive-care units: a cohort study. Lancet Infect Dis.

[4] Venier A-G, Leroyer C, Slekovec C, Talon D, Bertrand X (2014). Risk factors for *Pseudomonas aeruginosa* acquisition in intensive care units: a prospective multicentre study. J Hosp Infect.

[5] European Centre for Disease Prevention and Control (ECDC) (2019). Healthcare-Associated Infections in Intensive Care Units - Annual Epidemiological Report for 2017. https://www.ecdc.europa.eu/en/publications-data/healthcare-associated-infections-intensive-care-units-annual-epidemiological-1.

[6] Aujoulat F, Roger F, Bourdier A, Lotthé A, Lamy B (2012). From environment to man: genome evolution and adaptation of human opportunistic bacterial pathogens. Genes (Basel).

[7] Kaiser SJ, Mutters NT, DeRosa A, Ewers C, Frank U (2017). Determinants for persistence of *Pseudomonas aeruginosa* in hospitals: interplay between resistance, virulence and biofilm formation. *Eur J Clin Microbiol Infect Dis*.

[8] Mathee K, Narasimhan G, Valdes C, Qiu X, Matewish JM (2008). Dynamics of *Pseudomonas aeruginosa* genome evolution. Proc Natl Acad Sci USA.

[9] Baranovsky S, Royer G, Combaluzier S, Corne S, Romano-Bertrand S (2017). Monoclonal colonisation of ICU water network by *Pseudomonas aeruginosa*: residual infectious risk associated to water treated with antimicrobial filters. https://hal.umontpellier.fr/hal-02112211.

[10] Johansson E, Welinder-Olsson C, Gilljam M (2014). Genotyping of *Pseudomonas aeruginosa* isolates from lung transplant recipients and aquatic environment-detected in-hospital transmission. APMIS.

[11] Walker JT, Jhutty A, Parks S, Willis C, Copley V (2014). Investigation of healthcare-acquired infections associated with *Pseudomonas aeruginosa* biofilms in taps in neonatal units in Northern Ireland. J Hosp Infect.

[12] Gbaguidi-Haore H, Varin A, Cholley P, Thouverez M, Hocquet D (2018). A bundle of measures to control an outbreak of *Pseudomonas aeruginosa* associated with p-trap contamination. Infect Control Hosp Epidemiol.

[13] Petitjean M, Martak D, Silvant A, Bertrand X, Valot B (2017). Genomic characterization of a local epidemic *Pseudomonas aeruginosa* reveals specific features of the widespread clone ST395. Microb Genom.

[14] Varin A, Valot B, Cholley P, Morel C, Thouverez M (2017). High prevalence and moderate diversity of *Pseudomonas aeruginosa* in the U-bends of high-risk units in hospital. Int J Hyg Environ Health.

[15] Jeanvoine A, Meunier A, Puja H, Bertrand X, Valot B (2019). Contamination of a hospital plumbing system by persister cells of a copper-tolerant high-risk clone of *Pseudomonas aeruginosa*. Water Res.

[16] Virieux-Petit M, Hammer-Dedet F, Aujoulat F, Jumas-Bilak E, Romano-Bertrand S (2022). From copper tolerance to resistance in *Pseudomonas aeruginosa* towards patho-adaptation and hospital success. Genes.

[17] Faure E, Kwong K, Nguyen D (2018). *Pseudomonas aeruginosa* in chronic lung infections: how to adapt within the host?. Front Immunol.

[18] Cottalorda A, Leoz M, Dahyot S, Gravey F, Grand M (2020). Within-Host microevolution of *Pseudomonas aeruginosa* urinary isolates: a seven-patient longitudinal genomic and phenotypic study. Front Microbiol.

[19] Royer G, Virieux-Petit M, Aujoulat F, Hersent C, Baranovsky S (2024). Residual risk of *Pseudomonas aeruginosa* waterborne contamination in an intensive care unit despite the presence of filters at all water points-of-use. J Hosp Infect.

[20] Curran B, Jonas D, Grundmann H, Pitt T, Dowson CG (2004). Development of a multilocus sequence typing scheme for the opportunistic pathogen *Pseudomonas aeruginosa*. J Clin Microbiol.

[21] Société Française de Microbiologie (2019). Recommandations 2019 V.1.0 Janvier: Comité de l’antibiogramme de la Société Française de Microbiologie / EUCAST. https://www.sfm-microbiologie.org/2019/01/07/casfm-eucast-2019/.

[22] Abdouchakour F, Dupont C, Grau D, Aujoulat F, Mournetas P (2015). *Pseudomonas aeruginosa* and *Achromobacter sp*. clonal selection leads to successive waves of contamination of water in dental care units. Appl Environ Microbiol.

[23] Abdouchakour F, Aujoulat F, Licznar-Fajardo P, Marchandin H, Toubiana M (2018). Intraclonal variations of resistance and phenotype in Pseudomonas aeruginosa epidemic high-risk clone ST308: A key to success within a hospital?. Int J Med Microbiol.

[24] Corne P, Godreuil S, Jean-Pierre H, Jonquet O, Campos J (2005). Unusual implication of biopsy forceps in outbreaks of *Pseudomonas aeruginosa* infections and pseudo-infections related to bronchoscopy. J Hosp Infect.

[25] Croucher NJ, Page AJ, Connor TR, Delaney AJ, Keane JA (2015). Rapid phylogenetic analysis of large samples of recombinant bacterial whole genome sequences using Gubbins. Nucleic Acids Res.

[26] Bortolaia V, Kaas RS, Ruppe E, Roberts MC, Schwarz S (2020). ResFinder 4.0 for predictions of phenotypes from genotypes. J Antimicrob Chemother.

[27] Clausen P, Aarestrup FM, Lund O (2018). Rapid and precise alignment of raw reads against redundant databases with KMA. BMC Bioinformatics.

[28] Zankari E, Hasman H, Cosentino S, Vestergaard M, Rasmussen S (2012). Identification of acquired antimicrobial resistance genes. J Antimicrob Chemother.

[29] del Barrio-Tofiño E, López-Causapé C, Cabot G, Rivera A, Benito N (2017). Genomics and susceptibility profiles of extensively drug-resistant *Pseudomonas aeruginosa* isolates from Spain. Antimicrob Agents Chemother.

[30] López-Causapé C, Cabot G, Del Barrio-Tofiño E, Oliver A (2018). The versatile mutational resistome of *Pseudomonas aeruginosa*. Front Microbiol.

[31] Winsor GL, Griffiths EJ, Lo R, Dhillon BK, Shay JA (2016). Enhanced annotations and features for comparing thousands of *Pseudomonas* genomes in the *Pseudomonas* genome database. Nucleic Acids Res.

[32] Liu B, Zheng D, Zhou S, Chen L, Yang J (2022). VFDB 2022: a general classification scheme for bacterial virulence factors. Nucleic Acids Res.

[33] Alikhan N-F, Petty NK, Ben Zakour NL, Beatson SA (2011). BLAST Ring Image Generator (BRIG): simple prokaryote genome comparisons. BMC Genomics.

[34] Hua X, Liang Q, Deng M, He J, Wang M (2021). BacAnt: A Combination Annotation Server for Bacterial DNA sequences to identify antibiotic resistance genes, integrons, and transposable elements. Front Microbiol.

[35] Page AJ, Cummins CA, Hunt M, Wong VK, Reuter S (2015). Roary: rapid large-scale prokaryote pan genome analysis. Bioinformatics.

[36] Rissman AI, Mau B, Biehl BS, Darling AE, Glasner JD (2009). Reordering contigs of draft genomes using the Mauve aligner. Bioinformatics.

[37] Martak D, Meunier A, Sauget M, Cholley P, Thouverez M (2020). Comparison of pulsed-field gel electrophoresis and whole-genome-sequencing-based typing confirms the accuracy of pulsed-field gel electrophoresis for the investigation of local *Pseudomonas aeruginosa* outbreaks. J Hosp Infect.

[38] Spinler JK, Raza S, Thapa S, Venkatachalam A, Scott T (2022). Comparison of whole genome sequencing and repetitive element PCR for multidrug-resistant *Pseudomonas aeruginosa* strain typing. J Mol Diagn.

[39] Sanz-García F, Hernando-Amado S, Martínez JL (2018). Mutation-driven evolution of *Pseudomonas aeruginosa* in the presence of either ceftazidime or ceftazidime-avibactam. Antimicrob Agents Chemother.

[40] Snyder LA, Loman NJ, Faraj LA, Levi K, Weinstock G (2013). Epidemiological investigation of *Pseudomonas aeruginosa* isolates from a six-year-long hospital outbreak using high-throughput whole genome sequencing. Eurosurveillance.

[41] Turton JF, Wright L, Underwood A, Witney AA, Chan Y-T (2015). High-resolution analysis by whole-genome sequencing of an international lineage (Sequence Type 111) of *Pseudomonas aeruginosa* associated with metallo-carbapenemases in the United Kingdom. J Clin Microbiol.

[42] Willmann M, Bezdan D, Zapata L, Susak H, Vogel W (2015). Analysis of a long-term outbreak of XDR *Pseudomonas aeruginosa*: a molecular epidemiological study. J Antimicrob Chemother.

[43] Clark ST, Guttman DS, Hwang DM (2018). Diversification of Pseudomonas aeruginosa within the cystic fibrosis lung and its effects on antibiotic resistance. FEMS Microbiol Lett.

[44] Wang K, Chen Y-Q, Salido MM, Kohli GS, Kong J-L (2017). The rapid *in vivo* evolution of *Pseudomonas aeruginosa* in ventilator-associated pneumonia patients leads to attenuated virulence. Open Biol.

[45] Chen Y, Jia H, Liang Y, Zhang H, Che S (2020). Structural characterization of the *Pseudomonas aeruginosa* dehydrogenase AtuB involved in citronellol and geraniol catabolism. Biochem Biophys Res Commun.

[46] Falagas ME, Athanasaki F, Voulgaris GL, Triarides NA, Vardakas KZ (2019). Resistance to fosfomycin: mechanisms, frequency and clinical consequences. Int J Antimicrob Agents.

[47] Segev-Zarko L-A, Kapach G, Josten M, Klug YA, Sahl H-G (2018). Deficient lipid A remodeling by the arnB gene promotes biofilm formation in antimicrobial peptide susceptible *Pseudomonas aeruginosa*. Biochemistry.

[48] Rossi E, La Rosa R, Bartell JA, Marvig RL, Haagensen JAJ (2021). *Pseudomonas aeruginosa* adaptation and evolution in patients with cystic fibrosis. Nat Rev Microbiol.

[49] Valot B, Guyeux C, Rolland JY, Mazouzi K, Bertrand X (2015). What it takes to be a *Pseudomonas aeruginosa*? the core genome of the opportunistic pathogen updated. PLoS One.

[50] Subedi D, Vijay AK, Kohli GS, Rice SA, Willcox M (2018). Comparative genomics of clinical strains of *Pseudomonas aeruginosa* strains isolated from different geographic sites. Sci Rep.

[51] Pimentel BE, Moreno-Sánchez R, Cervantes C (2002). Efflux of chromate by *Pseudomonas aeruginosa* cells expressing the ChrA protein. FEMS Microbiol Lett.

[52] Zhao H, Clevenger AL, Coburn PS, Callegan MC, Rybenkov VV (2022). Condensins are essential for *Pseudomonas aeruginosa* corneal virulence through their control of lifestyle and virulence programs. Mol Microbiol.

[53] Brovedan MA, Cameranesi MM, Limansky AS, Morán-Barrio J, Marchiaro P (2020). What do we know about plasmids carried by members of the *Acinetobacter* genus?. *World J Microbiol Biotechnol*.

[54] Janice J, Agyepong N, Owusu-Ofori A, Govinden U, Essack SY (2021). Carbapenem Resistance determinants acquired through novel chromosomal integrations in extensively drug-resistant *Pseudomonas aeruginosa*. Antimicrob Agents Chemother.

[55] McLean MM, Chang Y, Dhar G, Heiss JK, Johnson RC (2013). Multiple interfaces between a serine recombinase and an enhancer control site-specific DNA inversion. Elife.

[56] Chichón G, López M, de Toro M, Ruiz-Roldán L, Rojo-Bezares B (2023). Spread of *Pseudomonas aeruginosa* ST274 clone in different niches: resistome, virulome, and phylogenetic relationship. *Antibiotics (Basel)*.

[57] Hayward C, Ross KE, Brown MH, Whiley H (2020). Water as a source of antimicrobial resistance and healthcare-associated infections. Pathogens.

[58] Hu Z, Yang L, Liu Z, Han J, Zhao Y (2023). Excessive disinfection aggravated the environmental prevalence of antimicrobial resistance during COVID-19 pandemic. Sci Total Environ.

[59] Caughlan RE, Sriram S, Daigle DM, Woods AL, Buco J (2009). Fmt Bypass in *Pseudomonas aeruginosa* causes induction of MexXY Efflux Pump Expression. Antimicrob Agents Chemother.

[60] Teixeira P, Tacão M, Alves A, Henriques I (2016). Antibiotic and metal resistance in a ST395 *Pseudomonas aeruginosa* environmental isolate: a genomics approach. Mar Pollut Bull.

[61] Jurado-Martín I, Sainz-Mejías M, McClean S (2021). *Pseudomonas aeruginosa*: an audacious pathogen with an adaptable arsenal of virulence factors. Int J Mol Sci.

[62] Moradali MF, Ghods S, Rehm BHA (2017). *Pseudomonas aeruginosa* lifestyle: a paradigm for adaptation, survival, and persistence. Front Cell Infect Microbiol.

[63] Petitjean M, Juarez P, Meunier A, Daguindau E, Puja H (2021). The rise and the fall of a *Pseudomonas aeruginosa* endemic lineage in a hospital. Microb Genom.

[64] Kung VL, Ozer EA, Hauser AR (2010). The accessory genome of *Pseudomonas aeruginosa*. Microbiol Mol Biol Rev.

[65] Kawalek A, Kotecka K, Modrzejewska M, Gawor J, Jagura-Burdzy G (2020). Genome sequence of *Pseudomonas aeruginosa* PAO1161, a PAO1 derivative with the ICEPae1161 integrative and conjugative element. BMC Genomics.

[66] Vasquez-Rifo A, Veksler-Lublinsky I, Cheng Z, Ausubel FM, Ambros V (2019). The *Pseudomonas aeruginosa* accessory genome elements influence virulence towards *Caenorhabditis elegans*. Genome Biol.

[67] Hao X, Li X, Pal C, Hobman J, Larsson DGJ (2017). Bacterial resistance to arsenic protects against protist killing. *Biometals*.

[68] Hao X, Lüthje F, Rønn R, German NA, Li X (2016). A role for copper in protozoan grazing - two billion years selecting for bacterial copper resistance. Mol Microbiol.

[69] Muchesa P, Leifels M, Jurzik L, Hoorzook KB, Barnard TG (2017). Coexistence of free-living amoebae and bacteria in selected South African hospital water distribution systems. Parasitol Res.

[70] Thomas V, Herrera-Rimann K, Blanc DS, Greub G (2006). Biodiversity of amoebae and amoeba-resisting bacteria in a hospital water network. Appl Environ Microbiol.

[71] Botelho J, Grosso F, Peixe L (2019). Antibiotic resistance in *Pseudomonas aeruginosa* - Mechanisms, epidemiology and evolution. Drug Resist Updat.

[72] Balasubramanian D, Kumari H, Mathee K (2015). *Pseudomonas aeruginosa* AmpR: an acute-chronic switch regulator. Pathog Dis.

[73] Gifford DR, Furió V, Papkou A, Vogwill T, Oliver A (2018). Identifying and exploiting genes that potentiate the evolution of antibiotic resistance. *Nat Ecol Evol*.

[74] Castanheira M, Deshpande LM, Costello A, Davies TA, Jones RN (2014). Epidemiology and carbapenem resistance mechanisms of carbapenem-non-susceptible *Pseudomonas aeruginosa* collected during 2009-11 in 14 European and Mediterranean countries. J Antimicrob Chemother.

[75] Lister PD, Wolter DJ, Hanson ND (2009). Antibacterial-resistant *Pseudomonas aeruginosa*: clinical impact and complex regulation of chromosomally encoded resistance mechanisms. Clin Microbiol Rev.

[76] Pelegrin AC, Palmieri M, Mirande C, Oliver A, Moons P (2021). *Pseudomonas aeruginosa*: a clinical and genomics update. FEMS Microbiol Rev.

[77] Li X-Z, Poole K (2001). Mutational analysis of the OprM outer membrane component of the MexA-MexB-OprM multidrug efflux system of *Pseudomonas aeruginosa*. J Bacteriol.

