# Supplementary material for: Adaptive evolution of Pseudomonas aeruginosa ST299 population colonizing a hospital copper water network over a 2.5-year period
Source: Microb Genom. 2026 Jan 27;12(1):001585. doi: 10.1099/mgen.0.001585 (PMC12847981; doi:10.1099/mgen.0.001585)
Supplement: Uncited Fig. S1. [file mgen-12-01585-s001.pdf]

Supplementary data :

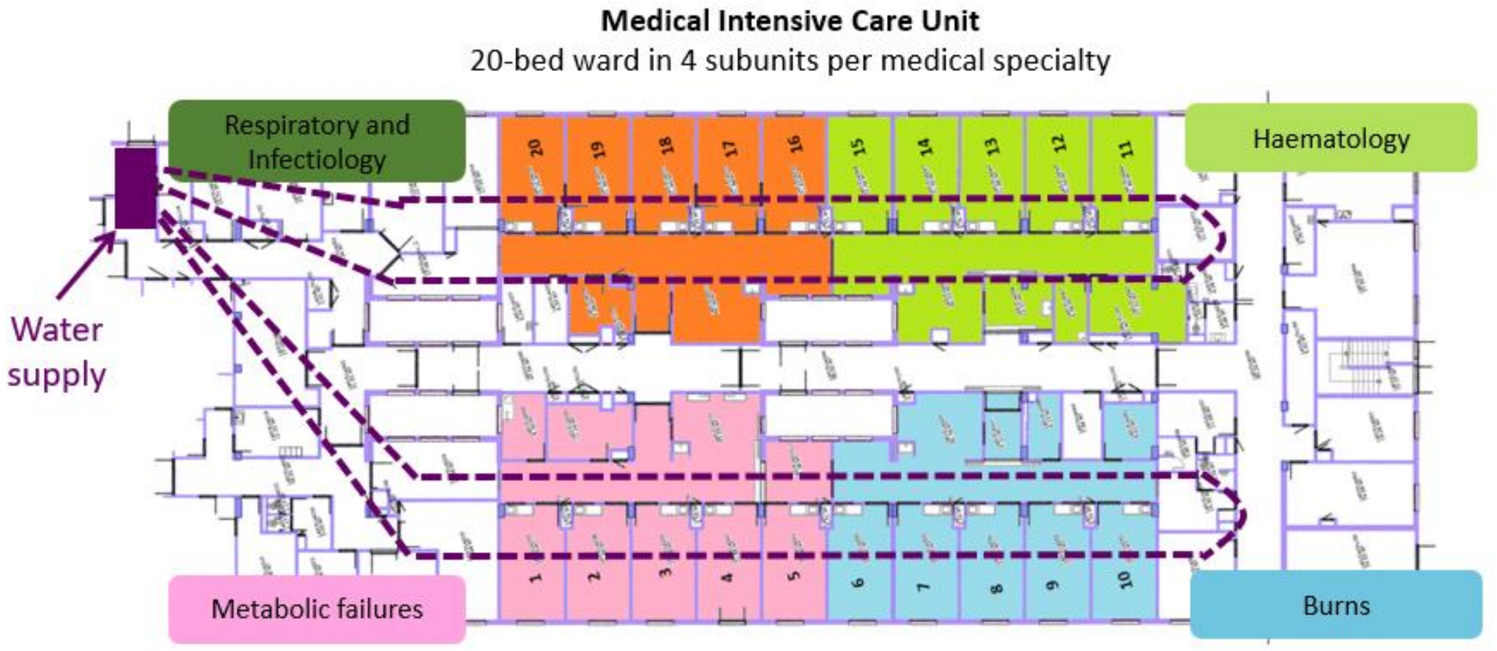

<u>Figure S1</u> : Organization of the water network in the intensive care unit

The water network is composed of two distinct supply loops represented by the purple dotted ovals. Each subunit is highlighted in a different color and have 5 individual rooms and a central space. Each bedroom and each central space are equipped with a terminal water point.

Table S1: **Characteristics of PA waterborne isolates collected from the water network of the medical ICU of the University Hospital of Montpellier and the PA clinical isolates responsible for colonization or infection in patients hospitalized in the medical ICU**

Name of isolate	Accession numbers	Type of sample (waterborne or clinical)	Collection date	Collection site (subunit or patient)	Type of water use point (CS or room) or sub-unit of hospitalization	ST	PFGE profile	Clade (based on core-SNP analysis with ref LTZ1)	cg-SNP profile
LTZ1	SRS20121925 GCA_037049625.1	waterborne	03/03/2015	Pink subunit	CS	299	A	A	1
LTZ7	SRS20121926	waterborne	03/03/2015	Pink subunit	Room 1	299	A	A	1
LTZ11	SRS20121937	waterborne	03/03/2015	Orange subunit	Room 17	299	A	A	1
LTZ17	SRS20121950	waterborne	03/03/2015	Blue subunit	CS	299	A	A	1
LTZ23	SRS20121960	waterborne	03/03/2015	Blue subunit	Room 9	299	A	A	1
LTZ25	SRS20121971	waterborne	03/03/2015	Green subunit loop	CS	299	A	A	1
LTZ31	SRS20121982	waterborne	03/03/2015	Green subunit	Room 13	299	A	A	1
MGU1	SRS20121993 GCA_037049585.1	waterborne	11/05/2015	Pink subunit	CS	2685			
MGU9	SRS20122004	waterborne	11/05/2015	Orange subunit	CS	299	Not done	A	1
MGU11	SRS20122015	waterborne	11/05/2015	Orange subunit	Room 17	299	Not done	A	2
MGU23	SRS20121927	waterborne	11/05/2015	Blue subunit	Room 9	299	Not done	A	1
MGU25	SRS20121929	waterborne	11/05/2015	Green subunit	CS	299	Not done	A	1
MGU31	SRS20121930	waterborne	11/05/2015	Green subunit	Room 13	299	Not done	A	1
MJK1	SRS20121931	waterborne	26/05/2015	Pink subunit	CS	2685			
MJK7	SRS20121932	waterborne	26/05/2015	Pink subunit	Room 1	299	Not done	A	1
MJK11	SRS20121933	waterborne	26/05/2015	Orange subunit	Room 17	299	Not done	A	1
MJK23	SRS20121934	waterborne	26/05/2015	Blue subunit	Room 9	299	Not done	A	3
MJK31	SRS20121935	waterborne	26/05/2015	Green subunit	Room 13	299	Not done	A	1
NDB7	SRS20121938	waterborne	20/08/2015	Orange subunit	CS	299	Not done	A	1
NDB9	SRS20121936	waterborne	20/08/2015	Orange subunit	Room 17	299	Not done	A	4
NDB15	SRS20121939	waterborne	20/08/2015	Blue subunit	Room 6	299	Not done	A	1
NDB23	SRS20121942	waterborne	20/08/2015	Green subunit	Room 13	299	Not done	A	1
NFL1	SRS20121940	waterborne	04/09/2015	Pink subunit	CS	2685			

NFL3	SRS20121941	waterborne	04/09/2015	Pink subunit	Room 1	299	Not done	A	1
NFL7	SRS20121943	waterborne	04/09/2015	Orange subunit	CS	2685			
NFL9	SRS20121945	waterborne	04/09/2015	Orange subunit	Room 17	299	Not done	A	1
NFL17	SRS20121946	waterborne	04/09/2015	Blue subunit	Room 9	299	Not done	A	1
NFL19	SRS20121947	waterborne	04/09/2015	Green subunit	CS	299	Not done	A	1
NFL23	SRS20121948	waterborne	04/09/2015	Green subunit	Room 13	299	Not done	A	1
NUA1	SRS20121949	waterborne	06/11/2015	Pink subunit	CS	299	A	A	1
NUA5	SRS20121952	waterborne	06/11/2015	Pink subunit	Room 1	299	A	A	1
NUA9	SRS20121951	waterborne	06/11/2015	Orange subunit	Room 17	299	A	A	1
NUA17	SRS20121954	waterborne	06/11/2015	Blue subunit	Room 9	299	A	A	5
OSU1	SRS20121953	waterborne	19/02/2016	Pink subunit	CS	299	A	A	1
OSU5	SRS20121955	waterborne	19/02/2016	Pink subunit	Room 1	299	A	A	1
OSU9	SRS20121956	waterborne	19/02/2016	Orange subunit	Room 17	299	A	A	1
OSU17	SRS20121957	waterborne	19/02/2016	Blue subunit	Room 9	299	A	A	1
OSU23	SRS20121958	waterborne	19/02/2016	Green subunit	Room 13	299	A	A	1
OXK1	SRS20121959	waterborne	07/03/2016	Pink subunit	CS	299	A	A	1
OXK5	SRS20121961	waterborne	07/03/2016	Pink subunit	Room 1	299	A	A	1
OXK7	SRS20121962	waterborne	07/03/2016	Orange subunit	CS	299	A	A	6
OXK9	SRS20121963	waterborne	07/03/2016	Orange subunit	Room 17	299	A	A	1
OXK13	SRS20121944	waterborne	07/03/2016	Blue subunit	CS	299	A	A	1
OXK17	SRS20121964	waterborne	07/03/2016	Blue subunit	Room 9	299	A	A	1
OXK19	SRS20121965	waterborne	07/03/2016	Green subunit	CS	299	A	A	1
OXK23	SRS20121966	waterborne	07/03/2016	Green subunit	Room 13	299	A	A	1
SDK1	SRS20121967	waterborne	08/11/2016	Pink subunit	CS	299	Not done	A	1
SDK5	SRS20121968	waterborne	08/11/2016	Pink subunit	Room 1	299	Not done	A	1
SDK7	SRS20121969	waterborne	08/11/2016	Orange subunit	CS	299	Not done	A	1
SDK9	SRS20121970	waterborne	08/11/2016	Orange subunit	Room 17	299	Not done	A	1
SDK13	SRS20121972	waterborne	08/11/2016	Blue subunit	CS	299	Not done	A	7
SDK15	SRS20121973	waterborne	08/11/2016	Blue subunit	Room 6	299	Not done	A	1
SDK21	SRS20121974	waterborne	08/11/2016	Green subunit	CS	299	Not done	A	1
SDK23	SRS20121975	waterborne	08/11/2016	Green subunit	Room 13	299	Not done	A	1
TGT1	SRS20121976	waterborne	17/03/2017	Pink subunit	CS	299	A	A	1
TGT5	SRS20121978	waterborne	17/03/2017	Pink subunit	Room 1	299	A	A	1
TGT7	SRS20121977	waterborne	17/03/2017	Orange subunit	CS	299	A	A	1
TGT9	SRS20121979	waterborne	17/03/2017	Orange subunit	Room 17	299	A	A	1

TGT13	SRS20121980	waterborne	17/03/2017	Blue subunit	CS	299	A	A	1
TGT17	SRS20121981	waterborne	17/03/2017	Blue subunit	Room 9	299	A	A	1
TGT19	SRS20121983	waterborne	17/03/2017	Green subunit	CS	299	A	A	1
TGT23	SRS20121984	waterborne	17/03/2017	Green subunit	Room 13	299	A	A	1
TXA5	SRS20121985	waterborne	02/06/2017	Pink subunit	Room 1	299	A	A	1
TXA9	SRS20121986	waterborne	02/06/2017	Orange subunit	Room 17	299	A	A	1
TXA13	SRS20121987	waterborne	02/06/2017	Blue subunit	CS	299	A	A	1
TXA17	SRS20121988	waterborne	02/06/2017	Blue subunit	Room 9	299	A	A	8
TXA23	SRS20121989	waterborne	02/06/2017	Green subunit	Room 13	299	A	A	1
UHR1	SRS20121990	waterborne	20/07/2017	Pink subunit	CS	299	Not done	A	1
UHR7	SRS20121991	waterborne	20/07/2017	Orange subunit	CS	299	Not done	A	1
UHR9	SRS20121992	waterborne	20/07/2017	Orange subunit	Room 17	299	Not done	A	1
UHR17	SRS20121994	waterborne	20/07/2017	Blue subunit	Room 9	299	Not done	A	1
UHR19	SRS20121995 GCA_037049605.1	waterborne	20/07/2017	Green subunit	CS	274			
UHR21	SRS20121996	waterborne	20/07/2017	Green subunit	Room 13	299	Not done	A	9
167-7	SRS20121998	clinical	30/06/2015	patient 1 (rectal carriage)	Subunit pink (ICU metabolic)	299	A	A	14
167-6	SRS20121997	clinical	06/07/2015	patient 1 (protected respiratory sampling)	Subunit pink (ICU metabolic)	299	A	A	13
157-3	SRS20121999	clinical	19/07/2015	patient 1 (expectoration)	Subunit pink (ICU metabolic)	299	A	A	14
157-23	SRS20122000	clinical	30/06/2015	patient 2 (blood culture)	Subunit orange (ICU infectiology)	299	A	A	15
157-22	SRS20122001	clinical	06/07/2015	patient 2 (expectoration)	Subunit orange (ICU infectiology)	299	A	A	15
157-21	SRS20122002	clinical	26/07/2015	patient 2 (bronchial aspiration)	Subunit orange (ICU infectiology)	299	A	A	15

167-28	SRS20122003	clinical	02/08/2015	patient 2 (catheter culture)	Subunit orange (ICU infectiology)	299	A	A	10
167-32	SRS20122005	clinical	09/11/2015	patient 3 (rectal carriage)	Subunit pink (ICU metabolic)	299	A	A	11
167-57	SRS20122006	clinical	08/12/2015	patient 4 (skin graft flap)	Subunit pink (ICU metabolic)	299	A	A	12
1796-5	SRS20122009	clinical	08/02/2016	patient 6 (rectal carriage)	Subunit pink (ICU metabolic)	299	A	A	16

Table S2: Geographical distribution of waterborne isolates

	Care subunits Pink (P) and Blue (B)	Care subunits Green (G) and Orange (O)
Water POU of CS	5 (B)<br>9 (P)	7 (O)<br>8 (G)
Water POU of room	12 (B : n°9)<br>8 (P : n°1)	12 (O : n°17)<br>11 (G : n°13)
Total isolates	35	38

<u>Table S3:</u> Characteristics of 16 isolates from the cystic fibrosis patient

<table>
<tr><th></th><th>Name of isolate</th><th>Accession numbers</th><th>Date of isolation</th><th>Profile rep-PCR multiplex</th></tr>
<tr><td rowspan="2">Sputum sample 1</td><td>III-69</td><td>SRS20122011/ GCA 037049525.1</td><td rowspan="2">26.05.2015</td><td rowspan="2">Profile 1</td></tr>
<tr><td>III-71</td><td>SRS20122010</td></tr>
<tr><td rowspan="7">Sputum sample 2</td><td>IV-62</td><td>SRS20122012</td><td rowspan="7">29.04.2016</td><td rowspan="7">Profile 2</td></tr>
<tr><td>IV-63</td><td>SRS20122013</td></tr>
<tr><td>IV-64</td><td>SRS20122014</td></tr>
<tr><td>IV-65</td><td>SRS20122016</td></tr>
<tr><td>IV-66</td><td>SRS20122018</td></tr>
<tr><td>IV-67</td><td>SRS20122017</td></tr>
<tr><td>IV-68</td><td>SRS20122019</td></tr>
<tr><td rowspan="3">Sputum sample 3</td><td>IV-74</td><td>SRS20122020</td><td rowspan="3">29.07.2016</td><td rowspan="2">Profile 3</td></tr>
<tr><td>IV-75</td><td>SRS20122021</td></tr>
<tr><td>IV-76</td><td>SRS20122022</td><td>Profile 4</td></tr>
<tr><td rowspan="4">Sputum sample 4</td><td>IV-85</td><td>SRS20122023</td><td rowspan="4">29.11.2016</td><td>Profile 4</td></tr>
<tr><td>IV-86</td><td>SRS20122024</td><td rowspan="3">Profile 5</td></tr>
<tr><td>IV-87</td><td>SRS20122025</td></tr>
<tr><td>IV-88</td><td>SRS20121928</td></tr>
</table>

<u>Table S4 :</u> Antibiotypes of *Pseudomonas aeruginosa* isolates of environmental and clinical origin

Inhibition diameters are expressed in mm. Interpretation is carried out according to the criteria of the Comité d'Antibiologie de la Société Française de Microbiologie (CA-SFM). The color code used here: red= isolate resistant to the antibiotic tested, green= isolate sensitive to the antibiotic tested.
* antibiotics designated by the Europoean Centre for Disease Prevention and Control (ECDC) for the determination of the antibiotic resistance in *Pseudomonas aeruginosa*

	Acide phosphonique	Polypeptides	Fluoroquinolones		Aminoglycosides				Cotrimoxazole	B-lactamines
Isolats	Fosfomycine (50 µg)	colistine (50 µg)	Ciprofloxacine* (5 µg)	Lévofloxacine (5 µg)	Netilmicine (30 µg)	Amikacine* (30 µg)	Gentamycine* (10 µg)	Tobramycine* (10 µg)	Triméthoprime/ sulfaméthoxazole (1,25µg/23,75µg)	Aztréonam* (30 µg)
LTZ1	39	23	35	28	23	23	19	22	0	30
LTZ7	36	22	33	27	20	22	19	23	0	31
NUA1	38	22	34	30	22	22	19	22	0	28
NUA17	37	21	35	28	23	23	19	22	0	28
OSU1	39	22	35	27	21	20	18	20	0	30
OXK1	37	20	35	30	21	22	18	24	0	30
SDK13	37	23	35	23	22	23	20	24	0	28
TGT1	37	21	36	27	22	23	19	22	0	29
TGT13	37	22	33	28	23	22	18	22	0	32
TXA13	37	21	33	23	21	19	15	19	0	28
157-3	29	21	37	17	22	22	18	21	0	17
167-6	28	20	35	29	23	21	17	20	0	21
167-7	30	23	31	18	21	22	18	22	0	14
157-21	25	22	32	27	22	18	17	20	0	17
157-22	24	21	37	28	21	22	19	22	0	14
157-23	23	21	37	28	21	22	17	22	0	28
167-32	27	21	35	30	21	20	17	20	0	25
167-57	21	22	28	21	22	24	19	22	0	22
1796-5	25	20	35	29	23	23	18	23	0	30

	B-lactamines												
	Penicillines						Céphalosporines					Carbapenemes	
Isolats	Ticarcilline* (75 µg)	Piperacilline* (30 µg)	Ticarcilline/ acide Clavulanique* (75 µg /10µg)	Piperacilline/ tazobatam* (30 µg /6µg)	Amoxicilline (25µg)	Amoxicilline/ acide clavulanique (20µg/10µg)	Ceftazidime* (30 µg)	Cefalexine (30µg)	Cefepime* (30 µg)	Ceftolozane/ Tazobactam* (30µg/10µg)	Cefotaxime (30 µg)	Imipeneme* (10 µg)	Meropeneme* (10 µg)
LTZ1	24	27	27	27	0	0	26	0	26	37	0	28	32
LTZ7	25	27	23	27	0	0	25	0	25	36	0	30	32
NUA1	22	24	22	27	0	0	24	0	24	36	0	31	32
NUA17	22	25	25	27	0	0	25	0	25	36	0	29	33
OSU1	24	29	24	27	0	0	23	0	25	37	0	30	33
OXK1	21	25	24	27	0	0	23	0	26	38	0	31	36
SDK13	23	25	25	27	0	0	28	0	27	39	0	32	35
TGT1	24	27	25	27	0	0	24	0	27	36	0	31	37
TGT13	21	24	24	28	0	0	21	0	26	39	0	32	36
TXA13	23	24	25	25	0	0	25	0	24	25	0	31	29
157-3	0	10	0	0	0	0	0	0	18	25	0	12	14
167-6	0	0	0	0	0	0	0	0	21	25	0	13	12
167-7	0	0	0	0	0	0	0	0	17	27	0	12	14
157-21	0	14	0	0	0	0	0	0	20	22	0	30	32
157-22	0	0	0	0	0	0	0	0	15	30	0	30	31
157-23	20	30	24	21	0	0	23	0	28	27	0	30	33
167-32	15	15	14	0	0	0	14	0	24	26	0	11	29
167-57	12	26	17	19	0	0	18	0	23	28	0	27	33
1796-5	21	30	23	22	0	0	23	0	27	27	0	29	32

		REF = LTZ1	MGU-11	MJK-23	NDB-9	NUA-17	OXK-7	SDK-13	TXA-17	UHR-21	167-28	167-32	167-57	167-6	167-7	157-23	1796-5
Contig	Position	Profil 1 (n=60)	Profile 2 (n=1)	Profile 3 (n=1)	Profile 4 (n=1)	Profile 5 (n=1)	Profile 6 (n=1)	Profile 7 (n=1)	Profile 8 (n=1)	Profile 9 (n=1)	Profile 10 (n=1)	Profile 11 (n=1)	Profile 12 (n=1)	Profile 13 (n=1)	Profile 14 (n=2)	Profile 15 (n=3)	Profile 16 (n=1)
4	159977	G										A					
6	5779	C									T					T	
6	44279	G													A		
6	79046	G															A
8	29667	C									A					A	
8	281148	G															T
9	229310	G									A					A	
12	213680	C										T					
13	8194	G								A							
14	49429	G						A									
18	191412	G										A					
19	21535	C									T					T	
19	93815	C					T										
27	33196	C									T					T	
31	8411	A											G				
33	72154	G		A													
36	137	T							C								
36	143	G							A								
36	78529	G	A														
36	90793	C				A											
39	9381	C												T			
40	43716	A									T						
45	42756	C													T		
46	5005	T			C												
50	4560	G	C														

VARIANT TYPES	PA01 (NC_002516)
MV	PA2886 atuA (expressed protein with apparent function in citronellol catabolism)
SV	PA4943 (probable GTP-binding protein)
MV	PA4975 NAD(P)H quinone oxidoreductase
MV	PA5002 dnpA (N-acetylase involved in persistence)
MV	PA3552 arnB
MV	PA3503 hypothetical protein
MV	PA3777 xseA (exodeoxyribonuclease VII large subunit)
MV	PA0691 phdA (prevent host death protein A)
MV	PA4931 dnaB
SG	PA1862 modB
MV	PA4450 murA (UDP-N acetylglucosamine 1 carboxyvinyltransferase)
Non-coding area	
SV	PA0563 conserved hypothetical protein
MV	PA4056 ribD (riboflavin specific deaminase/reductase)
SG	PA2246 bkdR (transcriptional regulator)
MV	PA0317 D-2 hydroxyglutarate dehydrogenase
SV	PA4133 cbb3-type
SV	PA4133 cbb3-type
SV	PA4201 ddlA (D-alanine-D-alanine ligase A)
Non-coding area	
SV	PA0181 (probable transcriptional regulator)
MV	PA1474 hypothetical protein
MV	PA0424 mexR (multidrug resistance operon repressor)
MV	PA5373 betB (betaine aldehyde dehydrogenase)
MV	PA2300 chiC (chitinase)

<u>Figure S2 :</u> Core-genome SNP (cgSNP) matrix of clade A. CgSNP alignment within the major clade A reports 25 bp with the 16 profiles.
The following abbreviations SV, MV and SG stand for Variant Synonym, Variant Missense and Stop Gain respectively associated with a low, moderate or high impact.

		REF = LTZ1	MGU-11	MJK-23	NDB-9	NUA-17	OXK-7	SDK-13	TXA-17	UHR-21		
Contig	Position	Profil 1 (n=60)	Profil 2 (n=1)	Profil 3 (n=1)	Profil 4 (n=1)	Profil 5 (n=1)	Profil 6 (n=1)	Profil 7 (n=1)	Profil 8 (n=1)	Profil 9 (n=1)	Type of mutation	PA01 (NC_002516)
13	8194	G								A	MV	PA4931 dnaB
14	49429	G						A			SG	PA1862 modB
19	93815	C					T				SV	PA0563 conserved hypothetical protein
33	72154	G		A							MV	PA0317 D-2 hydroxyglutarate dehydrogenase
36	137	T							C		SV	PA4133 cbb3-type
36	143	G							A		SV	PA4133 cbb3-type
36	78529	G	A								SV	PA4201 ddlA (D-alanine-D-alanine ligase A)
36	90793	C				A					Non coding area	
46	5005	T			C						MV	PA5373 betB (betaine aldehyde dehydrogenase)
50	4560	G	C								MV	PA2300 chiC (chitinase)

Figure S3 : Core-genome SNP (cgSNP) matrix of ICU-water clade A. CgSNP alignment within the major clade A reports 10 bp with the 9 profiles. *The following abbreviations SV, MV and SG stand for Variant Synonym, Variant Missense and Stop Gain respectively.*

Contig	Position	REF = III-69	III-71	IV-62	IV-63	IV-64	IV-65	IV-66	IV-67	IV-68	IV-74	IV-75	IV-76	IV-85	IV-86	IV-87	IV-88	Type of mutation	Genes impacted by SNP
2	11062	A	T		T				T		T	T					T	Non coding area	
3	29769	A			T				T		T						T	MV	Hypothetical protein
3	95314	A	C															MV	PA5015, aceE, Pyruvate deshydrogenase
4	81525	T			C													Non coding area	
6	1931	C														G		SV	PA0963, aspS, Aspartyl tRNA synthetase
7	192457	T								G								MV	PA1148, toxA, Exotoxin A precursor
10	93276	G	A	A		A			A			A		A	A	A	A	Non coding area	
10	93279	C	G	G		G			G			G		G	G	G	G	Non coding area	
10	93280	T	C	C		C			C			C		C	C	C	C	Non coding area	
10	93592	C													T			MV	PA1339, Glutamine transport ATP-binding protein GlnQ
11	39374	G													A			MV	**PA3545, algG, Alginate c5 mannuroman epimerase (periplasmic)**
14	56531	A								C								MV	PA0593, pdxA, pyridoxal phosphate biosynthetic protein
15	19053	A	C		C				C		C	C					C	MV	**PA0426, MexB, RND multidrug efflux transporter**
15	43057	**G**	C	C	C	C	C	C	C	C	C	C	C	C	C	C	C	MV	**PA0408, pilG, twitching motility protein (cytoplasmic)**
15	77071	C			T						T						T	SG	PA0371, putative Zn-dependent peptidase
17	240318	G		A			A											MV	PA1566, pauA3, Glutamylpolyamine synthetase
17	249134	C	A															SG	Hypothetical protein
18	142710	T											A					MV	PA2603.1, tRNA Ser
19	82887	T											G					MV	PA2144, glgP, glycogen phosphorylase
19	104920	C		G	G	G	G	G		G		G	G	G	G	G	G	MV	PA2124, probable deshydrogenase
19	104922	G		A	A	A	A	A		A		A	A	A	A	A	A	MV	PA2124, probable deshydrogenase
19	164724	G	A		A				A		A	A					A	MV	PA2098, probable esterase, deacetylase
19	187634	T											A					MV	PA2078, (7S,10S)-hydroperoxyde diol synthase
19	281712	A	G	G													G	Non coding area	
21	149502	C							T									SG	PA3839, probable sodium/sulfate symporter
22	10602	G															A	MV	**PA3878, narX, two component sensor**
23	31720	T	C															MV	Hypothetical protein
23	37593	T			G													MV	Hypothetical protein
24	30256	C			A													MV	PA3713, spdH, spermidine dehydrogenase
24	112929	G													A			SV	hypothetical protein
29	21407	G							A									SV	PA4592, probable outer membrane protein precursor
33	38062	G				A												SG	**PA0763, mucA, anti sigma factor**
36	43203	C								T								MV	Hypothetical protein
39	28636	C														G		MV	Hypothetical protein
41	66110	G	A		A				A		A	A					A	SV	PA2232, pslB
42	17346	G	C		C	C	C	C	C	C		C	C	C	C	C	C	MV	PA1497, probable transporter
42	17350	C	G		G	G	G	G	G	G		G	G	G	G	G	G	MV	PA1497, probable transporter
42	17351	G	C		C	C	C	C	C	C		C	C	C	C	C	C	MV	PA1497, probable transporter
44	104506	C		T		T	T	T						T	T	T		SG	**PA2960, pilZ, type IV fimbrial biogenesis protein**
47	96539	G					C											MV	PA5278, dapF, diaminopimelate epimerase
47	96541	C					A											MV	PA5278, dapF, diaminopimelate epimerase
48	27036	A								T			T					MV	**PA4526, pilB, type IV fimbrial biogenesis protein**
53	26790	T			C												C	Non coding area	
55	1667	A	G	G	G	G	G	G	G	G	G	G	G	G	G	G	G	SG	**PA4218, ampP**
57	8152	C											A					Non coding area	
58	22749	G											A					Non coding area	

Figure S4 : Core-genome SNP (cgSNP) matrix of clade B (CF strains). CgSNP alignment within the major clade B reports 46 bp with the 16 profiles. *The following abbreviations SV, MV and SG stand for Variant Synonym, Variant Missense and Stop Gain respectively. Patho-adaptive mutations are shown in red, and mutations concerning genes known to be involved in regulating the mutational resistome in blue.*

CHR	POS	REF (LTZ1)	157_21	157_22	157_23	157_3	167_28	167_32	167_57	167_6	167_7	1796_5	Type of mutation	Product
contig_0004	159977	G						A					MV	**PA2886 atuA (expressed protein with apparent function in citronellol catabolism)**
contig_0006	5779	C	T	T	T		T						SV	PA4943 (probable GTP-binding protein)
contig_0006	44279	G				A					A		MV	PA4975 NAD(P)H quinone oxidoreductase
contig_0006	79046	G										A	MV	PA5002 dnpA (N-acetylase involved in persistence)
contig_0008	29667	C	A	A	A		A						MV	**PA3552 arnB**
contig_0008	160493	C		G	G	G	G	G	G		G	G	SV	PA3417 (Pyruvate dehydrogenase E1 component subunit alpha)
contig_0008	281148	G										T	MV	PA3503 hypothetical protein
contig_0009	229310	G	A	A	A		A						MV	PA3777 xseA (exodeoxyribonuclease VII large subunit)
contig_0012	213680	C						T					MV	PA0691 phdA (prevent host death protein A)
contig_0013	205983	C					A						MV	PA2546 (ring cleaving dioxygenase)
contig_0015	42784	G					C						Non coding area	
contig_0018	191412	G											MV	**PA4450 murA (UDP-N acetylglucosamine 1 carboxyvinyltransferase)**
contig_0019	21535	C	T	T	T		T						Non coding area	
contig_0027	33196	C	T	T	T		T						MV	PA4056 ribD (riboflavin specific deaminase/reductase)
contig_0027	89695	C		T			T						MV	**PA4109 ampR (HTH-type transcriptional activator)**
contig_0030	35175	T	C	C			C						Non coding area	
contig_0031	8411	A							G				SG	PA2246 bkdR (transcriptional regulator)
contig_0039	9381	C								T			SV	PA0181 (probable transcriptional regulator)
contig_0040	43716	A					T						MV	PA1474(hypothetical protein)
contig_0040	66867	C	A		A		A						MV	PA1497 (urea transporter)
contig_0043	41875	G					C						Non coding area	
contig_0045	42756	C				T					T		MV	**PA0424 mexR (multidrug resistance operon repressor)**

Figure S5 : Core-genome SNP (cgSNP) matrix of ICU-clinical-associated strains of clade A. CgSNP alignment was of 22 bp. *The following abbreviations SV, MV and SG stand for Variant Synonym, Variant Missense and Stop Gain respectively. Patho-adaptive mutations are shown in red, and mutations concerning genes known to be involved in regulating the mutational resistome in blue.*

Supplementary data: Elimination of a methodological artefact

The genomic stability of ST299-PA during 2.5 years despite variations in the pressures exerted on its niche (repeated shocks of different kinds, changes in practices) (Royer et al. 2024) contrasts with the literature that describes the genome of PA as polymorph and flexible (Moradali, Ghods, et Rehm 2017; Jurado-Martín, Sainz-Mejías, et McClean 2021). Such a surprising result required excluding methodological biases.

First, compared to a cg/wgMLST approach, a cgSNP-based phylogeny offers higher resolution for intra-ST studies and highly related isolates (Cunningham et al. 2017; Miro et al. 2019). Nevertheless, several critical elements may impact the performance of the SNP call but the choice of bacterial pipeline and reference genome are major. Snippy pipeline is one of the most efficient method (Bush 2021), allowing to minimize false positive results easily (Bush 2021). Because PA

is a "recombinogenic" bacterium, the choice of reference genome has a critical effect on the phylogenetic relationships inferred from SNP calling, with suboptimal choice of reference genome resulting in mapping errors in the phylogeny. It is crucial that the chosen reference is closely related to the genomes being compared to increase SNP calling performance and reliability, hence the choice of LTZ1 (corresponding to the first waterborne isolate available in 3 March 2015 83 days after the water flow was turned on) as the reference. Moreover, to improve the PROKKA annotation of genes presenting SNPs, they were blasted in the genomic database PseudoCAP dedicated to PA.

Beside diversification by SNP, PA genome evolutes by recombination events that could blur phylogeny and provoke small- to large-size rearrangements (Darch et al. 2015) . The lack of SNP high-density areas, confirmed by Gubbins algorithm suggest the absence of major recombination events within the clade A (Croucher et al. 2015). Moreover, in coherence with macro-restriction patterns in PFGE, multiple comparison of the genomes via BLAST and MAUVE showed the absence of large-scale insertion or deletion. The use of several complementary bio-informatic tools validates data from Illumina's short-read which appears robust for our dataset despite no use of long-read sequencing.

Genome polymorphism and flexibility are involved in a main trait of the species PA which is the high diversity of variant populations displaying diverse profiles of antimicrobial resistance (AMR) and virulence factors. The stability of resistome and resistance patterns observed in ICU ST299-PA over 2.5 years was remarkable. To describe this resistome and its evolution, both ARG and point mutations mediating AMR (Cabot et al. 2016; López-Causapé et al. 2018) were search by two approaches : i) Resfinder software, for which excellent agreement between *in silico* and *in vitro* predicted phenotypes was found for most antimicrobial/species combinations (Bortolaia et al. 2020); ii) the KmerResistance software (Clausen, Aarestrup, et Lund 2018) that detected without prior genome assembly by splitting the reads into k-mer and mapping them to reference databases. Both approaches showed the low variability in resistome among ICU ST299-PA. This is confirmed by similar resistance phenotypes among water isolates whatever the date of isolation.

1. Royer G, Virieux-Petit M, Aujoulat F, Hersent C, Baranovsky S, Hammer-Dedet F, et al. Residual risk of Pseudomonas aeruginosa waterborne contamination in intensive care unit despite the presence of filters on all water points-of-use. J Hosp Infect. 3 mai 2024;S0195-6701(24)00153-1.

2. Moradali MF, Ghods S, Rehm BHA. Pseudomonas aeruginosa Lifestyle: A Paradigm for Adaptation, Survival, and Persistence. Front Cell Infect Microbiol. 2017;7:39.

3. Jurado-Martín I, Sainz-Mejías M, McClean S. Pseudomonas aeruginosa: An Audacious Pathogen with an Adaptable Arsenal of Virulence Factors. Int J Mol Sci. 18 mars 2021;22(6):3128.

4. Cunningham SA, Chia N, Jeraldo PR, Quest DJ, Johnson JA, Boxrud DJ, et al. Comparison of Whole-Genome Sequencing Methods for Analysis of Three Methicillin-Resistant Staphylococcus aureus Outbreaks. J Clin Microbiol. juin 2017;55(6):1946-53.

5. Miro E, Rossen JWA, Chlebowicz MA, Harmsen D, Brisse S, Passet V, et al. Core/Whole Genome Multilocus Sequence Typing and Core Genome SNP-Based Typing of OXA-48-Producing Klebsiella pneumoniae Clinical Isolates From Spain. Front Microbiol. 2019;10:2961.

6. Bush SJ. Generalizable characteristics of false-positive bacterial variant calls. Microb Genom. 4 août 2021;7(8):000615.

7. Darch SE, McNally A, Harrison F, Corander J, Barr HL, Paszkiewicz K, et al. Recombination is a key driver of genomic and phenotypic diversity in a Pseudomonas aeruginosa population during cystic fibrosis infection. Sci Rep. 12 janv 2015;5:7649.

8. Croucher NJ, Page AJ, Connor TR, Delaney AJ, Keane JA, Bentley SD, et al. Rapid phylogenetic analysis of large samples of recombinant bacterial whole genome sequences using Gubbins. Nucleic Acids Res. 18 févr 2015;43(3):e15.

9. Cabot G, López-Causapé C, Ocampo-Sosa AA, Sommer LM, Domínguez MÁ, Zamorano L, et al. Deciphering the Resistome of the Widespread Pseudomonas aeruginosa Sequence Type 175 International High-Risk Clone through Whole-Genome Sequencing. Antimicrob Agents Chemother. 21 nov 2016;60(12):7415-23.

10. López-Causapé C, Cabot G, del Barrio-Tofiño E, Oliver A. The Versatile Mutational Resistome of Pseudomonas aeruginosa. Front Microbiol. 6 avr 2018;9:685.

11. Bortolaia V, Kaas RS, Ruppe E, Roberts MC, Schwarz S, Cattoir V, et al. ResFinder 4.0 for predictions of phenotypes from genotypes. J Antimicrob Chemother. 11 août 2020;75(12):3491-500.

12. Clausen PTLC, Aarestrup FM, Lund O. Rapid and precise alignment of raw reads against redundant databases with KMA. BMC Bioinformatics. 29 août 2018;19:307.
